# IGF2BP3-mediated translation in cell protrusions promotes cell invasiveness and metastasis of pancreatic cancer

**DOI:** 10.18632/oncotarget.2257

**Published:** 2014-07-25

**Authors:** Keisuke Taniuchi, Mutsuo Furihata, Kazuhiro Hanazaki, Motoaki Saito, Toshiji Saibara

**Affiliations:** ^1^ Department of Pharmacology, Kochi Medical School, Kochi University, Nankoku, Kochi, Japan; ^2^ Department of Pathology, Kochi Medical School, Kochi University, Nankoku, Kochi, Japan; ^3^ Department of Surgery, Kochi Medical School, Kochi University, Nankoku, Kochi, Japan; ^4^ Department of Gastroenterology and Hepatology, Kochi Medical School, Kochi University, Nankoku, Kochi, Japan

**Keywords:** RNA-binding protein, pancreatic cancer, cell invasion and metastasis, stress granule, local translation, cell protrusion

## Abstract

Pancreatic cancers are aggressive because they are highly invasive and highly metastatic; moreover, effective treatments for aggressive pancreatic cancers are lacking. Here, we report that IGF2BP3 promoted the invasiveness and metastasis of pancreatic cancers through locally translated IGF2BP3-bound transcripts. In neural cells, transcripts sorted into cytoplasmic RNA granules are transported to dendrites and translated in these dendrites, thereby mediating long-term synaptic plasticity; however, such cytoplasmic RNA granules are not known to contribute to the progression of pancreatic cancer. We show evidence that IGF2BP3 and IGF2BP3-bound transcripts are localized in cytoplasmic RNA granules that accumulate in membrane protrusions of pancreatic cancer cells. Specific IGF2BP3-bound transcripts—*ARF6* and *ARHGEF4*—that are preferentially translated in membrane protrusions induce further formation of membrane protrusions; consequently, IGF2BP3 promotes cell invasiveness and tumor metastasis. Our results provide insight into the link between regulation of localized translation in cell protrusions and the invasiveness and metastasis of pancreatic cancers. New therapies that prevent local translation in cell protrusions may hold significant clinical promise.

## INTRODUCTION

RNA-binding proteins are involved in multiple aspects of RNA maturation, RNA turnover, translation, and movement of transcripts throughout the cell. IGF2BP1, IGF2BP2, IGF2BP3 (insulin-like growth factor-2 mRNA-binding proteins 1, 2, and 3) belong to a conserved family of RNA-binding proteins [1, 2, 3, 4]. The gene encoding IGF2BP3 was initially identified as the gene encoding the KH-domain-containing RNA-binding protein that is overexpressed in pancreatic ductal adenocarcinoma (PDAC) [4]. IGF2BP3 is not present in normal pancreatic tissues, benign lesions of the pancreas, or chronic pancreatitis [5]. IGF2BP3 enhances the formation of invadopodia (membrane protrusions that project into and digest the extracellular matrix) that accelerate migration of HeLa cells; specifically, IGF2BP3 prevents degradation of *CD44* messenger RNA (mRNA) in HeLa cells by associating with the 3′ untranslated region of this mRNA [6]. IGF2BP3 can also induce cell proliferation and invasiveness via post-transcriptional regulation of *IGF2*; the resulting IGF2 then activates oncogenic phosphatidylinositcol 3-kinase/mitogen-activated protein kinase (PI3K/MAPK) pathways in glioblastomas [7, 8]. Therefore, IGF2BP3 is likely to play a role in cell proliferation and migration via post-transcriptional regulation because it binds with mRNAs and assembles into protein-mRNA complexes.

We recently reported that intracellular CD24 binds with several RNA-binding proteins in cytoplasmic RNA granules called stress granules (SGs) of PDAC cells [9]. Many stressors induce SG formation, and these SGs contain mRNA, small ribosomal subunit proteins, and stress-dependent RNA-binding proteins that are involved in translation initiation or in mRNA degradation [10]. CD24 localized in SGs is transported to cell protrusions that are associated with cell migration [9]. In these protrusions, CD24 specifically associates with mRNAs that are targets of post-transcriptional regulation; CD24 thereby modulates the invasiveness and metastasis of PDAC cells by regulating the activity of Rho GTPases (Rac1 and RhoA) and a protein kinase C (PKCα) [11, 12, 13, 14]. Moreover, we found that intracellular CD24 may bind to IGF2BP3 [9]. This finding indicates that IGF2BP3 may modulate cell invasion and metastasis via a role in post-transcriptional regulation of specific target transcripts in PDAC cells.

Here, we found that cytoplasmic SGs containing IGF2BP3-mRNA complexes accumulated in membrane protrusions of PDAC cells. Further investigation revealed that IGF2BP3-bound mRNAs were subsequently and selectively translated in membrane protrusions; in turn, these locally translated proteins influenced formation of additional membrane protrusions and thereby increased the invasive and metastatic properties of the PDAC cells.

## RESULTS

### IGF2BP3 localizes in cell protrusion of migrating PDAC cells

**Figure 1:**
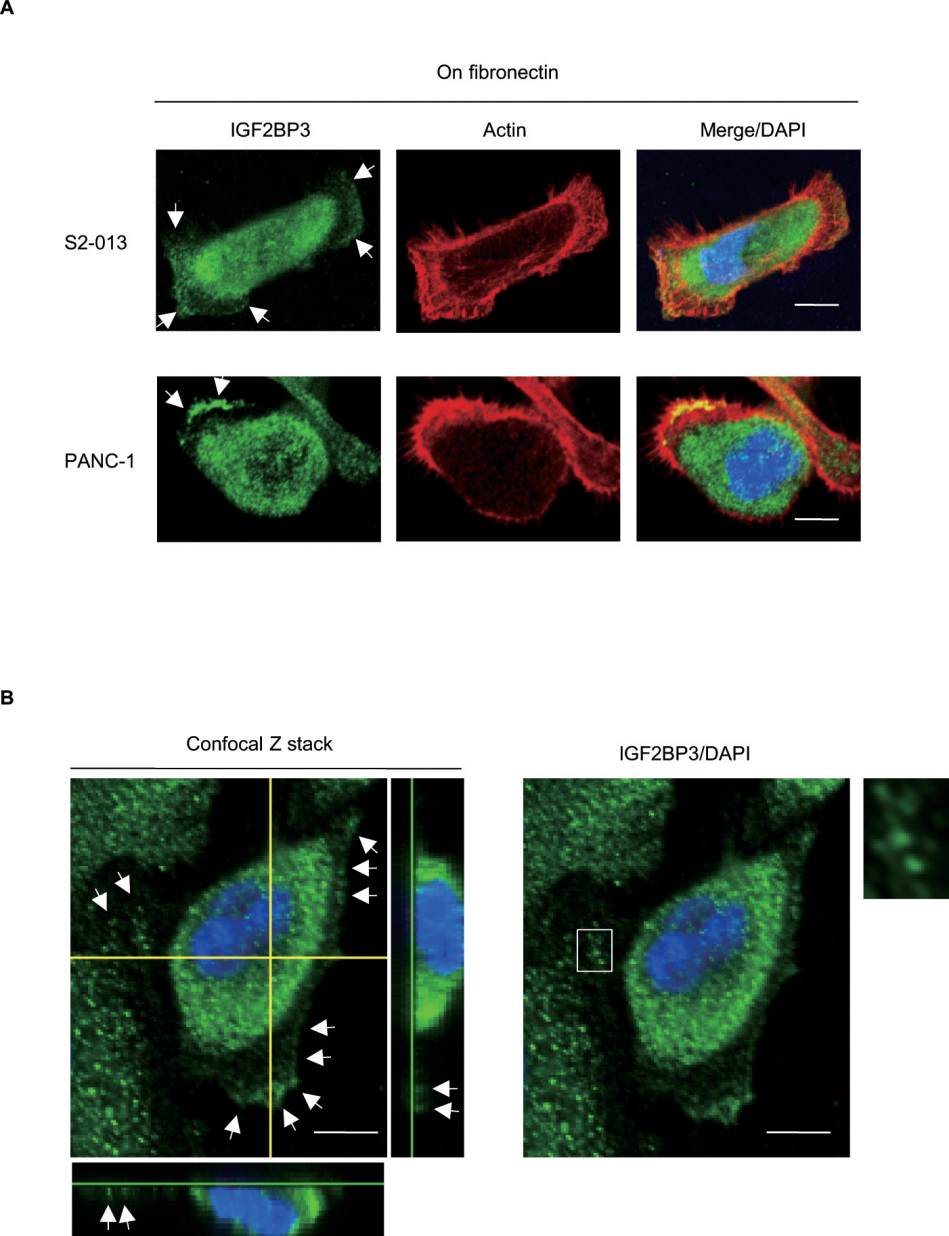
Distribution of IGF2BP3 in PDAC cells (A) S2-013 and PANC-1 cells were incubated on fibronectin and immunocytochemically labeled with anti-IGF2BP3 antibody (green). Actin filaments were labeled by phalloidin (red). Arrows, IGF2BP3 localized in cell protrusions. Bars, 10 μm. (B) Confocal Z stack shows and nuclear DAPI staining (blue) and IGF2BP3 (green) staining associated with granules in spreading S2-013 cells. Arrows, IGF2BP3 localized in cell protrusions. The white box indicates region shown in the enlarged image. The lower and light panels in the confocal Z stack show a vertical cross-section (yellow lines) through the cells. Bars, 10 μm.

We used immunocytochemistry to determine the subcellular localization of IGFBP3 in two types of cultured PDAC cells, moderately differentiated PDAC cells (line S2-013 [15]) and cells from a poorly differentiated PDAC line (PANC-1 [16]). S2-013 is a cloned subline of a PDAC cell line (SUIT-2) derived from a liver metastasis [15], and was obtained from Dr. T. Iwamura (Miyazaki Medical College, Miyazaki, Japan). Notably, when S2-013 cells that were initially in suspension attach to an immobilized fibronectin substrate, nascent membrane protrusions (*de novo* formation of actin patches at the cell periphery) form, and as these protrusions mature, they promote cell motility [13]. To investigate whether IGF2BP3 was localized in cell protrusions, fibronectin-stimulated cells were used. When S2-013 cells were cultured on fibronectin, cell spreading promoted accumulation of IGF2BP3 in membrane protrusions, which each had many peripheral actin structures (Figure 1A). Similarly, IGF2BP3 was accumulated in cell protrusions of fibronectin-stimulated PANC-1 cells (Figure 1A). Z stack panels showed that fibronectin-stimulated S2-013 cells exhibited intracellular expression of IGF2BP3 in cytoplasmic granules that were located in membrane protrusions (Figure 1B).

### Stable knockdown of IGF2BP3 reduces invasiveness and metastasis of S2-013 cells

**Figure 2:**
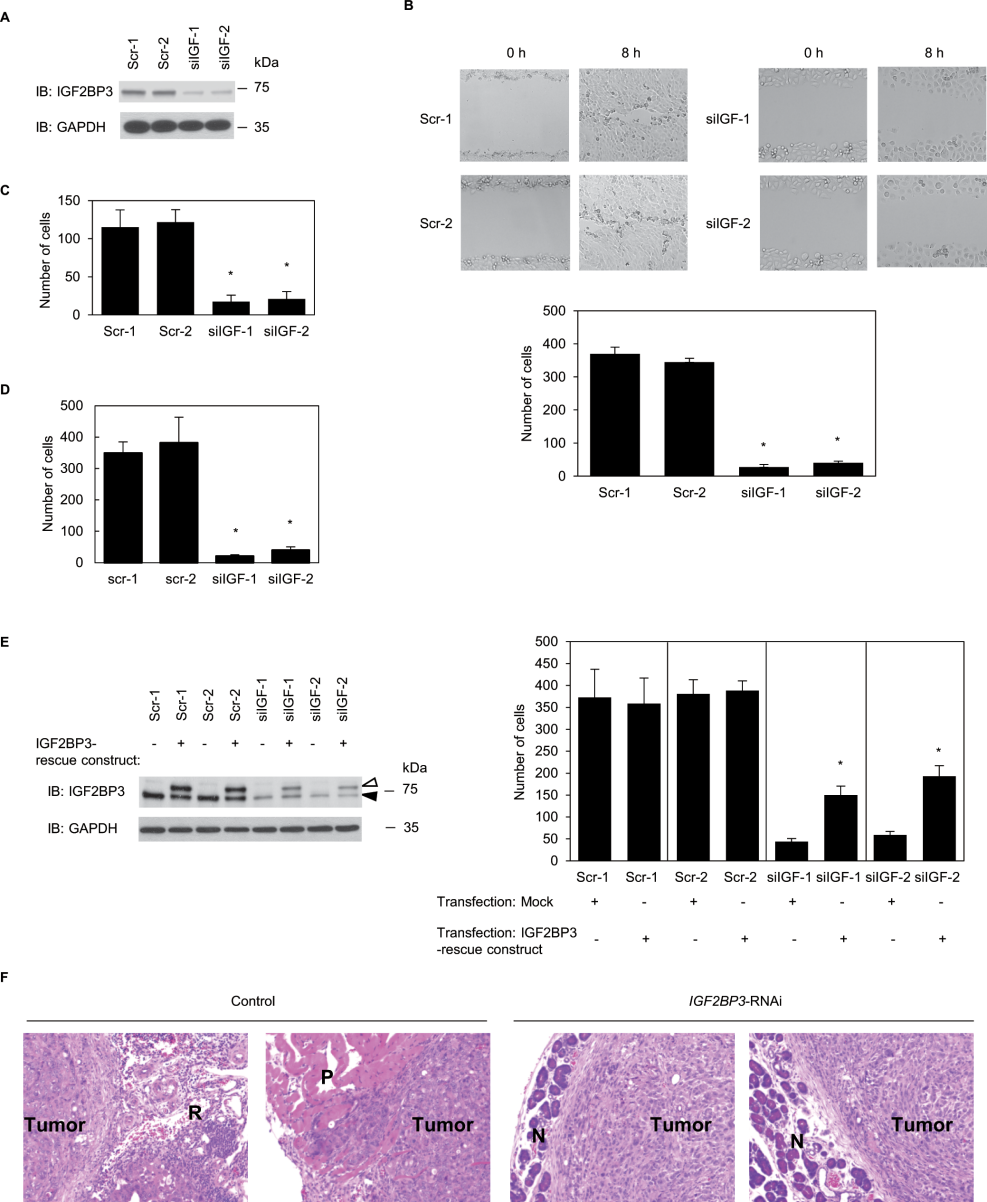
IGF2BP3 promotes cell motility and invasion in PDAC cell culture (A) Effect of *IGF2BP3*-siRNA in S2-013 cells. Western blots probed with anti-IGF2BP3 antibody show two S2-013 *IGF2BP3-RNAi* clones (siIGF-1-2) transfected with siRNA targeting *IGF2BP3* and two scrambled control-RNAi clones (Scr-1-2). (B) Confluent cell monolayers of control-RNAi S2-013 cells or *IGF2BP3*-RNAi S2-013 cells were wounded (upper panels). Cells that migrated into an initially cell-free wound were counted; specifically, cells in four defined areas per group per experiment were counted (lower panel). Data derive from three independent experiments. *Columns*, mean; *bars*, SD. *p < 0.001 compared to Scr-1 or Scr-2 (Student's *t*-test). (C) Control-RNAi or *IGF2BP3*-RNAi S2-013 cells were seeded into two-chamber motility chambers. Migrating cells in four fields per group were counted. Data derive from three independent experiments. *Columns*, mean; *bars*, SD. *p < 0.001 compared to Scr-1 or Scr-2 (Student's *t*-test). (D) Control-RNAi or *IGF2BP3*-RNAi S2-013 cells were seeded into Matrigel invasion chambers. Invading cells in four fields per group were counted. Data derive from three independent experiments. *Columns*, mean; *bars*, SD. *p < 0.001 compared to Scr-1 or Scr-2 (Student's *t*-test). (E) The mock control vector or myc-tagged IGF2BP3-rescue construct was transiently transfected into control-RNAi and *IGF2BP3*-RNAi cells; 48 h later, the two-chamber invasion assay was performed. Western blots probed with anti-IGF2BP3 antibody are shown in left panels. Closed arrow head, endogenous IGF2BP3; open arrow head, exogenous IGF2BP3. Invading cells in four fields per group were counted (right panels). Data derive from three independent experiments. *Columns*, mean; *bars*, SD. *p < 0.005 compared with corresponding siIGF-1 or siIGF-2 transfected mock vector (Student's *t*-test). (F) Hematoxylin and eosin staining of paraffin sections from xenograft, pancreatic tumors derived from control-RNAi S2-013 cells or *IGF2BP3-*RNAi S2-013 cells. Control-RNAi cells aggressively invaded surrounding pancreatic tissue in mice. *IGF2BP3*-RNAi cells were evident only at the capsular interface of tumor with the pancreas. R, retroperitoneal soft-tissue; P, peritoneal wall; N, normal pancreatic tissue. Original magnification: × 200.

To investigate whether IGF2BP3 affected cell motility and invasion, IGF2BP3 expression in S2-013 cells was suppressed by vector-based expression of an *IGF2BP3*- siRNA. To achieve substantial suppression of IGF2BP3, we established cell clones subject to RNA interference (RNAi) by expressing an *IGF2BP3*-siRNA. IGF2BP3 knockdown was confirmed on immunoblots (Figure 2A). Suppression of IGF2BP3 expression in S2-013 cells did not affect cell growth in an *in vitro* MTT assay (data not shown), but it did inhibit cell motility into a wounded area of confluent cultures (Figure 2B). In trans-well motility assays, motility of S2-013 cells was significantly lower in *IGF2BP3*-knockdown cells than in control cells (Figure 2C). In two-chamber invasion assays, *IGF2BP3*-RNAi S2-013 cells were significantly less invasive than the control-RNAi S2-013 cells (Figure 2D). We found that transfection of an IGF2BP3-rescue construct into *IGF2BP3*-RNAi S2-013 cells abrogated the changes to cell invasiveness caused by the *IGF2BP3*-RNAi (Figure 2E).

An orthotopic tumor challenge in nude mice was used to examine tumor invasiveness and metastasis. Incidence of regional invasion of retroperitoneum by PDAC cells was lower in mice injected with *IGF2BP3*-RNAi S2-013 cells than those injected with S2-013 control-RNAi cells; moreover, the *IGF2BP3*-RNAi cells did not form hepatic or lung metastases, but control-RNAi S2-013 cells did (Table 1). Control-RNAi S2-013 cells invaded pancreatic tissue throughout the entire organ; notably, borders between tumor lesions and intact normal pancreatic tissue were not obvious in the control-RNAi samples (data not shown). In contrast, each tumor derived from *IGF2BP3*-RNAi cells was largely encapsulated by host stromal cells and was apparently separated from normal pancreatic tissues (Figure 2F). In mice injected with control-RNAi S2-013 cells, the surface of the peritoneum was covered with a relatively thick layer of cancer cells, and cancer cells invaded muscular tunics (Figure 2F); however, in mice injected with *IGF2BP3*-RNAi cells, large areas of peritoneum were free of cancer cells (data not shown). These results indicated that IGF2BP3 specifically promoted PDAC cell invasion and metastasis and that during pancreatic tumorigenesis *in vivo* reduction in the amount of IGF2BP3 limited 1) tumor growth within the pancreas, 2) regional invasion of adjacent pancreatic tissue, and 3) metastasis to other organs.

**Table 1:** Metastatic potential of stable control S2-013 cells or IGF2BP3-RNAi cells *in vivo*

Cell line	Mice autopsied	Median tumor weight (g) (range)	Lung metastasis	Liver metastasis	Retroperitonem invasion
Scr-1	9	0.9 (0.6-1.8)	5/9	3/9	8/9
Scr-2	9	1.4 (0.6-2.0)	5/9	4/9	9/9
siIGF-1	10	1.0 (0.5-1.2)	0/10^a^	0/10^a^	5/10^a^
siIGF-2	9	1.3 (0.5-2.5)	0/9^a^	0/9^a^	3/9^a^

a p < 0.05 compared with Scr-1 or Scr-2 (Fisher's exact test)

### IGF2BP3 localizes in stress granules

**Figure 3:**
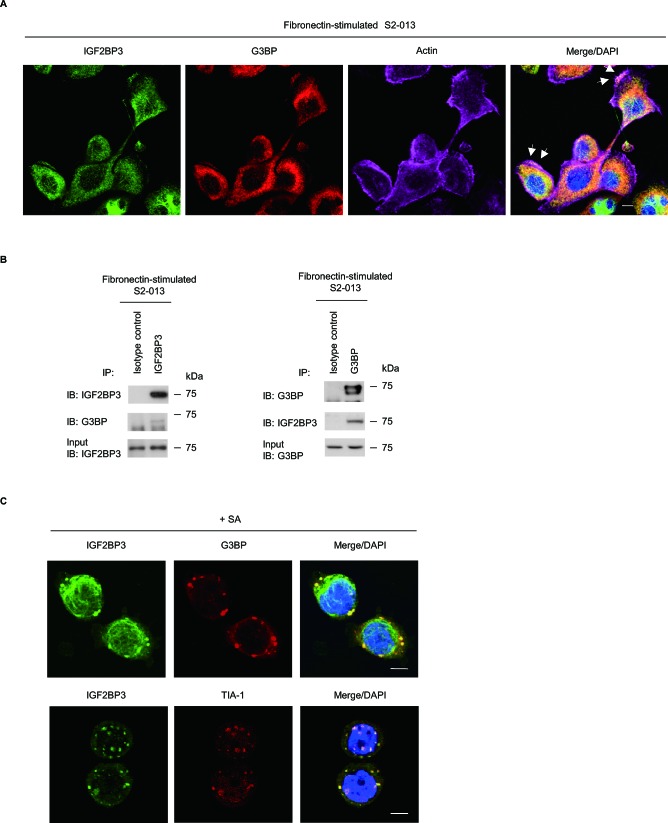
IGF2BP3 localizes in cytoplasmic SGs (A) S2-013 cells were incubated on fibronectin and stained with anti-IGF2BP3 (green) and anti-G3BP (red) antibodies. Actin filaments were labeled by phalloidin (violet). Arrows, IGF2BP3 colocalized with G3BP in cell protrusions. Blue, DAPI staining. Bar, 10 μm. (B) Immunoprecipitation of IGF2BP3 or G3BP from S2-013 cells cultured on fibronectin. Proteins in immunoprecipitates were examined on western blots probed with antibodies against IGF2BP3 and G3BP. Rabbit or mouse IgG isotype control antibody was used as an isotype control. (C) S2-013 cells were exposed to 500 μM SA for 30 min. Immunocytochemical staining with anti-IGF2BP3 antibody (green) and anti-G3BP or anti-TIA-1 (red) antibody are shown. Blue, DAPI staining. Bars, 10 μm.

The intracellular CD24 that associates with RNA-binding proteins in cytoplasmic SGs interacts with an SG marker G3BP and immunoprecipitaes with IGF2BP3 in PDAC cells [9]. SGs contain some 40S subunit ribosomal proteins and several types of translation initiation factors, which together represent stalled translation preinitiation complexes; SGs also contain several types of RNA-binding proteins [17]. To investigate whether IGF2BP3-containing granules were SGs, S2-013 cells cultured on fibronectin were double-labeled with anti-IGF2BP3 and anti-G3BP [18] antibodies. We found that IGF2BP3 colocalized with G3BP in granules in membrane protrusions in which peripheral actin structures were increased (Figure 3A). Cytoplasmic IGF2BP3 that localized in the cytoplasm of the cell bodies did not colocalize with G3BP. Furthermore, extracts from S2-013 cells that had been grown on fibronectin were subjected to immunoprecipitation (IP) with anti-IGF2BP3 or anti-G3BP; notably, IGF2BP3 co-immunoprecipitated along with G3BP (Figure 3B). To verify that IGF2BP3 was present in SGs, S2-013 cells were subjected to sodium arsenite (SA)-induced oxidative stress and then double-labeled with anti-IGF2BP3 and anti-G3BP or anti-TIA-1. SGs form in the cytoplasm of S2-013 cells when SA is added to complete medium [9]. TIA-1 functions as a translation repressor, and like G3BP, it localizes to SGs [19]. When S2-013 cells were treated with SA, IGF2BP3 that localized in cytoplasmic granules colocalized with G3BP and with TIA-1, but cytoplasmic IGF2BP3 that did not localized in SGs did not colocalize with G3BP or TIA-1 (Figure 3C). These data indicated that IGF2BP3 localized in SGs may function to regulate levels of certain mRNAs in membrane protrusions.

### Identification of IGF2BP3-bound transcripts

**Figure 4:**
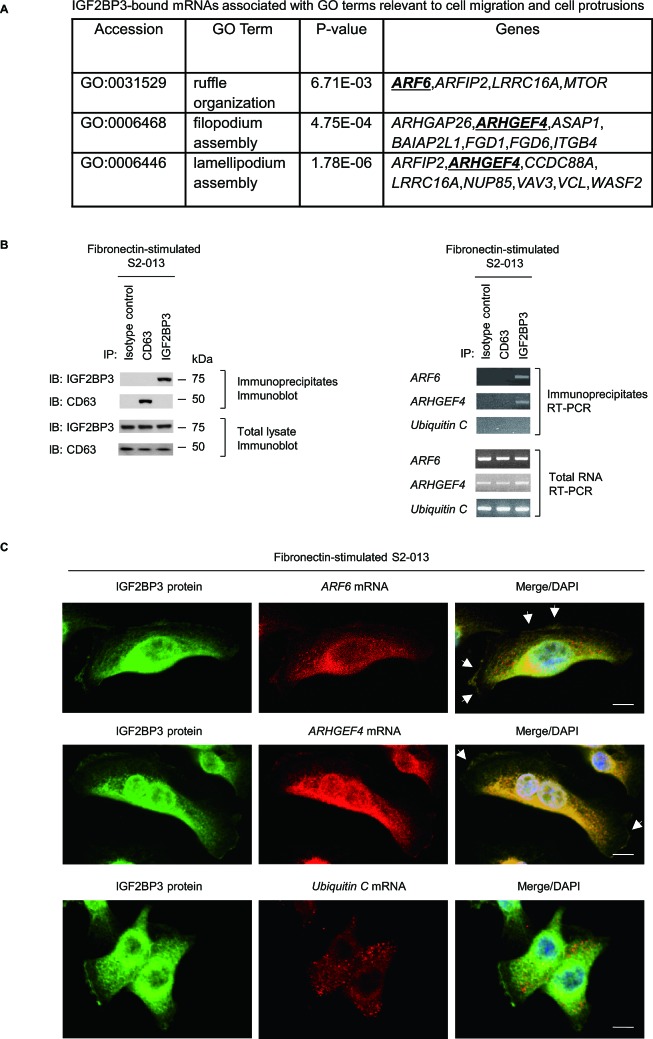
IGF2BP3 colocalizes with *ARF6* mRNA and *ARHGEF4* mRNA (A) Those IGF2BP3-bound transcripts that were identified in the RIP analysis and that are included in GO terms relevant to cell motility, invasiveness, and protrusions are shown. Underlines indicate *ARF6* and *ARHGEF4*. (B) The association between IGF2BP3 and *ARF6* mRNA or *ARHGEF4* mRNA in S2-013 cells cultured on fibronectin was tested via IGF2BP3-IP or control-IP and subsequent RT-PCR amplification of any *ARF6*, *ARHGEF4*, and *Ubiquitin C* in the immunoprecipitate (right panels). Proteins in immunoprecipitates were examined on western blots probed with antibodies against IGF2BP3 and CD63 (left panels). Rabbit IgG isotype control and anti-CD63 antibodies were used as negative controls for coimmunoprecipitation. (C) Colocalization of IGF2BP3 protein (green), and *ARF6* or *ARHGEF4* mRNA (red) in S2-013 cells cultured on fibronectin. *Ubiquitin C* mRNA was used as a negative control for colocalization. Arrows, mRNAs colocalized with IGF2BP3 in cell protrusions. Blue, DAPI staining. Bars, 10 μm.

To investigate whether RNA itself was present in IGF2BP3-containing granules and to identify any IGF2BP3-bound transcripts localized in these granules, we performed RNA immunoprecipitation with anti-IGF2BP3 and extracts from S2-013 cells that had been cultured on fibronectin; we then used next-generation sequencing to identify any mRNAs in the resultant immunoprecipitates (Figure S1A-D). The results of RIP assay are presented as log ratios in Table S1. We identified 2,826 RNAs that were significantly enriched in anti-IGF2BP3 immunoprecipitates relative to rabbit IgG isotype control immunoprecipitates (Table S1). The complete gene list derived from the 2,826 RNAs was uploaded onto the Gene Expression Omnibus Database http://www.ncbi.nlm.nih.gov/geo/ (GEO accession: GSE47597). To gain further insight into the biological functionalities of these IGF2BP3-bound mRNAs, the list of identified genes were subjected to gene ontology (GO) analysis focused on the GO category of “Biological Processes”. A larger number of GO terms matched the gene list (*P* < 10^−5^; Table S2), and this GO set was significantly enriched with cellular functions relevant to apoptosis, cell cycle, signal transduction, cell proliferation, cell adhesion, and cell migration. The transcripts that matched any GO term related to both cell migration and cell protrusion are listed in Figure 4A. We used RT-PCR to validate two of transcripts from this list; these IGF2BP3-bound mRNAs were ADP-ribosylation factor 6 (*ARF6*) and Rho guanine nucleotide exchange factor 4 (*ARHGEF4*). RT-PCR was performed on complexes immunoprecipitated with anti-IGF2BP3, rabbit IgG isotype control antibody, or anti-CD63 antibody; neither isotype control antibody nor anti-CD63 was expected to immunoprecipitate *ARF6* or *ARHGEF4* mRNA (Figure 4B). Both transcripts immunoprecipitated with anti-IGF2BP3, but neither transcript immunoprecipitated with isotype control antibody or anti-CD63.

Immunocytochemistry and RNA fluorescence *in situ* hybridization were used together to determine whether IGF2BP3 colocalized with each mRNA (*ARF6* and *ARHGEF4*) within cell protrusions of S2-013 cells cultured on fibronectin; each mRNA colocalized with IGF2BP3 in granules in the cytoplasm and those assembled within cell protrusions (Figure 4C). Control *ubiquitin C* mRNA did not colocalized with IGF2BP3 in fibronectin-stimulated S2-013 cells (Figure 4C). IGF2BP3 granules also accumulated in the perinuclear area; these granules were probably transported, along with the *ARF6* and *ARHGEF4* mRNAs, from this perinuclear area to cell protrusions. These results indicated that the granules that contained IGF2BP3 and IGF2BP3-bound mRNAs accumulated in cell protrusions.

### IGF2BP3 is associated with local translation in cell protrusions

**Figure 5:**
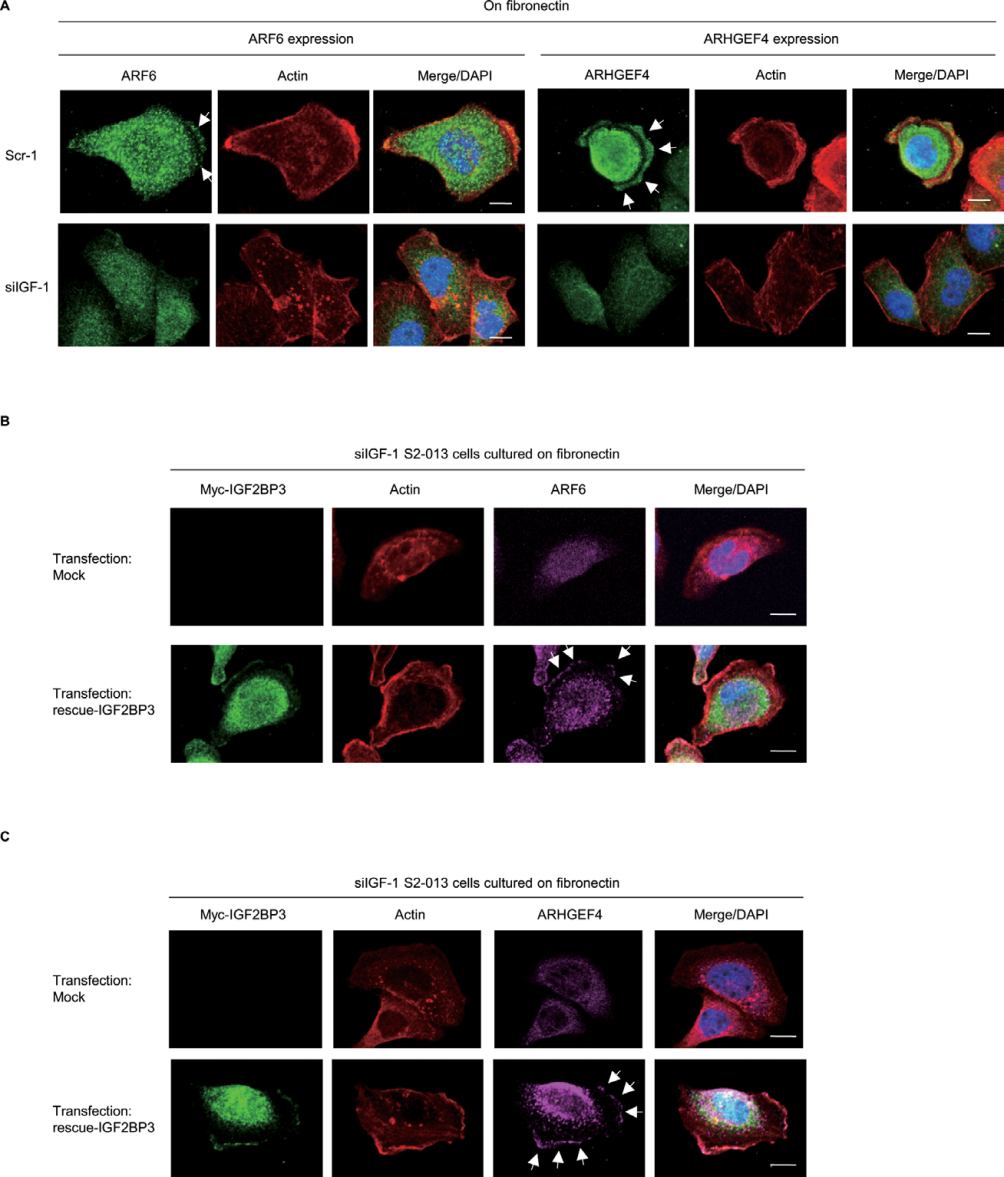
IGF2BP3-associated transcripts *ARF6* and *ARHGEF4* are translated in cell protrusions (A) Control-RNAi (Scr-1) S2-013 cells or *IGF2BP3*-RNAi (siIGF-1) S2-013 cells were incubated on fibronectin and stained with anti-ARF6 or anti-ARHGEF4 antibody (green). Actin filaments were labeled by phalloidin (red). Arrows, ARF and ARHGEF4 localized in cell protrusions. Blue, DAPI staining. Bars, 10 μm. (B) The myc-tagged IGF2BP3-rescue construct was transfected into *IGF2BP3*-RNAi S2-013 cells (siIGF-1). 48 h later, the cells were incubated on fibronectin. The cells were stained with antibodies against myc (green) and ARF6 (violet). Actin filaments were labeled by phalloidin (red). Arrows, ARF6 expression in cell protrusions. Blue, DAPI staining. Bars, 10 μm. (C) S2-013 cells treated as in (B) were stained with antibodies against myc (green) and ARHGEF4 (violet). Actin filaments were labeled by phalloidin (red). Arrows, ARHGEF4 expression in cell protrusions. Blue, DAPI staining. Bars, 10 μm.

We hypothesized that IGF2BP3-bound mRNAs accumulated in cell protrusions may be locally translated in the protrusions. Specifically, we used control-RNAi S2-013 cells, *IGF2BP3*-RNAi S2-013 cells, and immunocytochemistry to determine whether IGF2BP3 had a role in ARF6 or ARHGEF4 protein localization. All cells were cultured on fibronectin. ARF6 and ARHGEF4 were expressed in the cytoplasm and membrane protrusions of control-RNAi cells; notably, immunofluorescent signals from ARF6 and ARHGEF4 in cell protrusions were weaker in *IGF2BP3*-RNAi cells than in control-RNAi cells; in fact, the ARF6 and ARHGEF4 signals in *IGF2BP3*-RNAi cells were restricted to cytoplasm of cell bodies (Figure 5A). Interestingly, peripheral actin structures seemed to be decreased in *IGF2BP3*-knockdown cells, compared to control cells (Figure 5A). Transfection of an IGF2BP3-rescue construct renewed expression of ARF6 (Figure 5B) and of ARHGEF4 (Figure 5C) in membrane protrusions of *IGF2BP3*-RNAi S2-013 cells. These findings indicated that IGF2BP3 was associated with translational regulation of the transcripts for *ARF6* and *ARHGEF4* in these membrane protrusions.

### IGF2BP3 functions in forming cell protrusions

**Figure 6:**
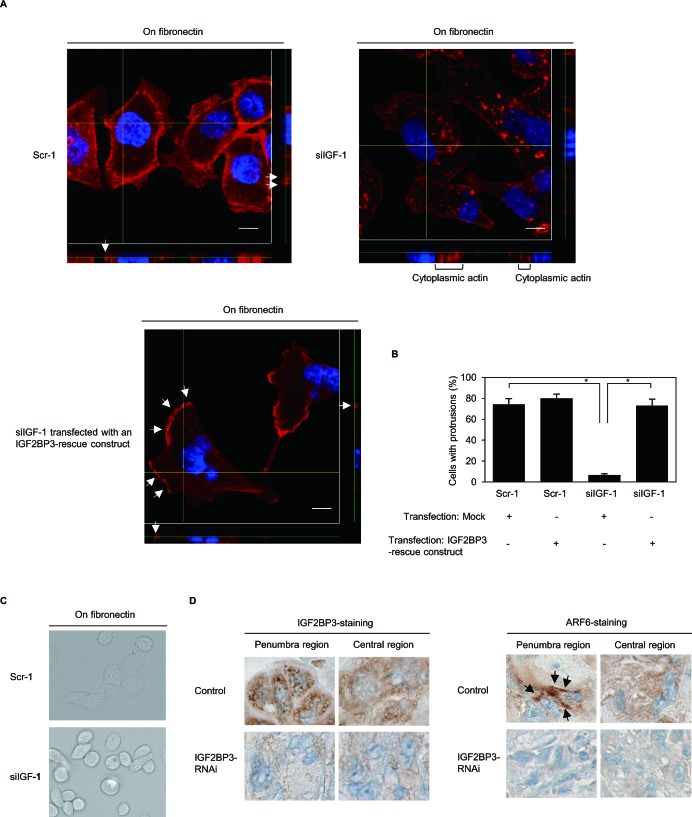
IGF2BP3 associates with forming cell protrusions (A) Confocal Z stack shows phalloidin-labeled peripheral actin structures (red) and DAPI-labeled nuclei (blue) in fibronectin-stimulated scrambled control-RNAi (Scr-1) S2-013 cells or *IGF2BP3*-RNAi (siIGF-1) S2-013 cells transfected with or without the myc-tagged IGF2BP3-rescue construct. Arrows, peripheral actin structures in cell protrusions. The lower and right panels in the confocal Z stack show a vertical cross-section (yellow lines) through the cells. Bars, 10 μm. (B) Quantification of data shown in Figure 6A; the values represent the number of cells with fibronectin-mediated cell protrusions in which peripheral actin structures were increased. All cells in four fields per group were scored. Data derive from three independent experiments. *Columns*, mean; *bars*, SD. *p < 0.001 compared with Scr-1 or siIGF-1 transfected mock vector (Student's *t*-test). (C) After scrambled control-RNAi (Scr-1) S2-013 cells or *IGF2BP3*-RNAi (siIGF-1) S2-013 cells were cultured on fibronectin for 4 h, the morphology of each cell was analyzed by phase-contact microscopy. (D) Immunohistochemical staining with anti-IGF2BP3 and anti-ARF6 antibodies in control or *IGF2BP3*-RNAi S2-013 primary pancreatic tumors in mice. Representative sections (× 400). Arrows, ARF6 localized near cell membranes.

Confocal microscopy was used to examine the 3-dimentional configurations of peripheral actin structures and cell protrusions in fibronectin-stimulated S2-013 cells. Peripheral actin structures (Figure 6A) and cell protrusions (Figure 6B) were less abundant in *IGF2BP3*-RNAi S2-013 cells than in control-RNAi S2-013 cells. Conversely, phalloidin-labeled actin structures were more abundant in the cytoplasm of the cell bodies of *IGF2BP3*-RNAi S2-013 cells than that of control-RNAi S2-013 cells (Figure 6A). Transfection of an IGF2BP3-rescue construct renewed peripheral actin structures in *IGF2BP3*-RNAi S2-013 cells (Figure 6A). Cell protrusions were significantly more abundant in *IGF2BP3*-RNAi S2-013 cells carrying an *IGF2BP3-*rescue construct than in *IGF2BP3*-RNAi S2-013 cells lacking this contruct (Figure 6B). Whereas parental control-RNAi S2-013 clones exhibited spindle-shaped cells and fibroblastic morphology, *IGF2BP3*-RNAi cells typically displayed a cobblestone-like, epithelial morphology (Figure 6C). These results indicated that IGF2BP3 drove rearrangement of peripheral actin to induce formation of additional membrane protrusions.

We analyzed the contribution of ARF6 to the IGF2BP3-mediated invasiveness and metastasis of PDAC cells. Nude mice were injected with control-RNAi S2-013 cells, and cytoplasmic granules containing IGF2BP3 were evident in the penumbra of each primary pancreatic tumor taken from such a mouse; moreover, ARF6 was enriched near cell membranes in the penumbra of each tumor (Figure 6D). For each tumor derived from control-RNAi S2-013 cells, cytoplasmic IGF2BP3-containing granules were less abundant in the central region than in the penumbra of the tumor; moreover, ARF6 mainly localized in the cytoplasm of these cancer cells (Figure 6D). In contrast, ARF6 levels in the cell membranes in the penumbra were lower in tumors derived from *IGF2BP3*-RNAi cells than in tumors derived from control-RNAi cells (Figure 6D). Notably, granular IGF2BP3 and membranous ARF6 are abundant only in the penumbra of control tumors, and *IGF2BP3*-knockdown decreased membranous ARF6 in the penumbra.

### ARF6 and ARHGEF4 have a role in the formation of cell protrusions

**Figure 7:**
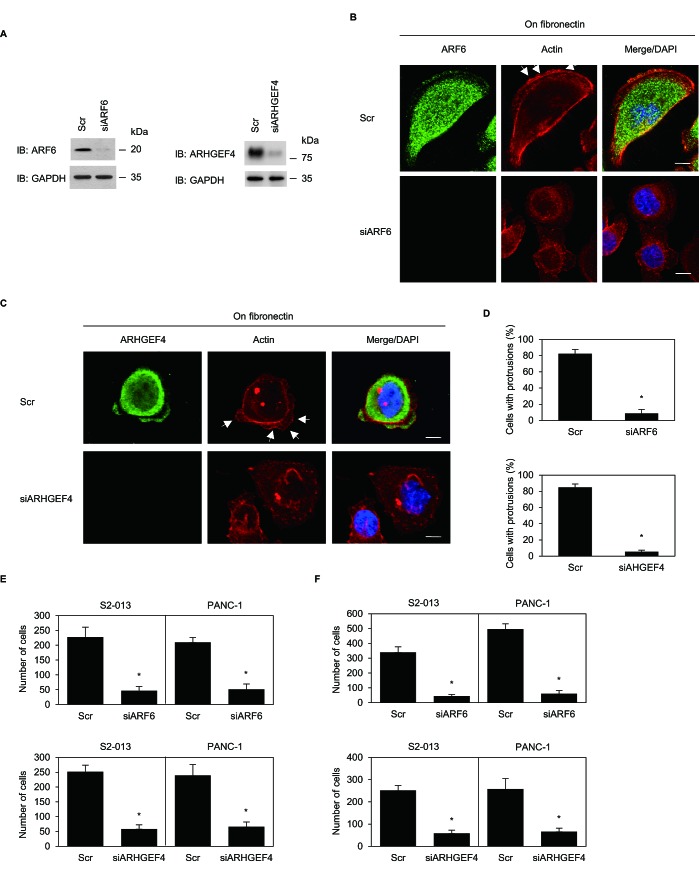
*ARF6* and *ARHGEF4* promote cell motility and invasion via forming cell protrusions (A) RNA oligonucleotides were transiently transfected into S2-013 cells; the siRNAs targeted *ARF6* (siARF6) or *ARHGEF4* (siARHGEF4); the negative control was a scrambled RNA (Scr). Western blot was performed using anti-ARF6 or anti-ARHGEF4 antibody. (B) Oligonucleotides were transiently transfected into S2-013 cells; the siRNA targeted *ARF6* (siARF6); the negative control was a scrambled RNA (Scr). S2-013 cells transfected with Scr or siARF6 were incubated on fibronectin, and cells were stained with anti-ARF6 antibody (green) and phalloidin (red). Arrows; peripheral actin structures in cell protrusions of Scr-transfected cells. Blue, DAPI staining. Bars, 10 μm. (C) Oligonucleotides were transiently transfected into S2-013 cells; the siRNA (siARHGEF4) targeted *ARHGEF4*. Scr- or siAHGEF4- transfected S2-013 cells were incubated on fibronectin, and cells were stained with anti-ARHGEF4 antibody (green) and phalloidin (red). Arrows; peripheral actin structures in cell protrusions of Scr-transfected cells. Blue, DAPI staining. Bars, 10 μm. (D) Quantification of data shown in Figure 7B and 7C, as described in Figure 6B. *Columns*, mean; *bars*, SD. *p < 0.001 compared with Scr-transfected controls (Student's *t*-test). (E, F) Oligonucleotides targeting *ARF6* or *ARHGEF4* or Scr was transiently transfected into S2-013 or PANC-1cells. The motility (E) and two-chamber invasion assays (F) were performed. Migrating cells in four fields per group were scored. Data derive from three independent experiments. *Columns*, mean; *bars*, SD. *p < 0.001 compared with Scr-transfected control (Student's *t*-test).

To determine whether ARF6, ARHGEF4, or both participated in the induction of membrane protrusions, we analyzed peripheral actin structures in membrane ruffles of control-RNAi, *ARF6*-RNAi, and *ARHGEF4*-RNAi S2-013 cells cultured on fibronectin. Based on western blot data, 72 h after transfection, expression of ARF6 or ARHGEF4 was markedly higher in control siRNA-transfected S2-013 cells than in *ARF6* siRNA-transfected or *ARHGEF4* siRNA-transfected, respectively (Figure 7A). Confocal microscopy revealed that *ARF6*- or *ARHGEF4*-knockdown in S2-013 decreased peripheral actin structures (Figure 7B for *ARF6*-knockdown and Figure 7C for *ARHGEF4*-knockdown). Furthermore, *ARF6*- or *ARHGEF4*-knockdown in S2-013 cells significantly inhibited fibronectin-mediated formation of membrane protrusions (Figure 7D). These results indicated that ARF6 and ARHGEF4 played a role in forming these membrane protrusions.

### ARF6 and ARHGEF4 promote motility and invasiveness of PDAC cells

Trans-well motility and Matrigel invasion assays and siRNA-mediated knockdown were used to examine the effect of ARF6 and ARHGEF4 on motility and invasiveness of S2-013 and PANC-1 cells; ARF6 and ARHGEF4 were highly expressed in both cell types. In trans-well motility assays, motility of S2-013 cells and of PANC-1 cells was significantly lower in *ARF6*- or *ARHGEF4*-knockdown cells than in control cells (Figure 7E). In two-chamber invasion assays, invasiveness of S2-013 and of PANC-1 cells was significantly lower in *ARF6*- or *ARHGEF4*-knockdown cells than in control cells (Figure 7F). These results indicated that ARF6 and ARHGEF4 promoted motility and invasiveness of PDAC cells.

## DISCUSSION

Here, we describe a newly discovered function for IGF2BP3; specifically, we found that 1) IGF2BP3 bound a specific set of mRNAs and 2) these IGF2BP3-mRNA complexes assembled into cytoplasmic RNA granules that are analogous to SGs. These IGF2BP3-bound mRNAs accumulated in cell protrusions and were translated in the protrusions, where they probably promoted the invasiveness and metastasis of the PDAC cells.

In neural cells, local translation is one efficient means of localizing proteins to specific synapses within a single cell; local translation allows selected synapses among the thousands of synapses in one neuron to autonomously control synaptic strength and efficacy [20]; such local translation requires translocation of particular mRNAs from cell bodies to dendrites [21]. In PDACs, we found that *ARF6* and *ARHGEF4* mRNAs were two of many transcripts enriched in IGF2BP3 immunoprecipitate. ARF6 is localized at invadopodia of cultured breast cancer cells and plays pivotal roles in actin-cytoskeletal remodeling at the cell periphery; moreover, suppression of ARF6 effectively blocks the invasive activities of these breast cancer cells—including the formation of invadopodia, localized matrix degradation, and Matrigel transmigration [22, 23]. The adenomatous polyposis coli (APC) protein enhances the activity of ARHGEF4 and stimulates ARHGEF4-mediated processes in MDCK cells; these processes include cell flattening, membrane ruffling, and lamellipodia formation [24]. We found that IGF2BP3-bound *ARF6* and *ARHGEF4* mRNAs were assembled in cell protrusions. *IGF2BP3*-knockdown did not decrease the expression of the ARF6 or ARHGEF4 that was localized in the cytoplasm of the cell bodies; however, *IGF2BP3*-knockdown did decrease the expression of the ARF6 and the ARHGEF4 that occurred in cell protrusions of fibronectin-stimulated S2-013 cells. Notably, the restoration of IGF2BP3 expression restored the expression of ARF6 and ARHGEF4 in cell protrusions. These findings indicated that IGF2BP3 was associated with translational regulation only in cell protrusions. Moreover, the finding that IGF2BP3 that localized in the cytoplasm of the cell body did not bind SG marker proteins (G3BP and TIA-1) indicated that the function of granular IGF2BP3 assembled in the protrusions might have been different from that of cytoplasmic IGF2BP3. The molecular differences (sequence and post-translational modifications) between granular IGF2BP3 and cytoplasmic IGF2BP3 are currently unknown. The restoration of IGF2BP3 expression in cell protrusions also induced formation of cell protrusions (Figure 6B) and knockdown of ARF6 and ARHGEF4 inhibited generation of cell protrusions (Figure 7D). These findings indicated that ARF6 and ARHGEF4 locally translated in cell protrusions had a role in the formation of cell protrusions. Local signaling events may play a role in mRNA release from cytoplasmic IGF2BP1-containing RNA granules [3]. The molecular mechanisms by which particular mRNAs dissociated from IGF2BP3-containing RNA granules and undergo local translation are important subjects for future study; nevertheless, our findings from this study that pertain to local translation in cell protrusions in PDAC cells are probably relevant to local translation in the dendrites of neural cells.

CD24 and G3BP-containing SGs assembled in the protrusions of PDAC cells contribute to post-transcriptional regulation of a specific set of mRNAs that, in turn, promote the invasiveness and metastasis of PDAC cells [9, 12]. Similarly, we found that IGF2BP3 and G3BP-containing SGs accumulated in cell protrusions could contribute to promotion of invasion and metastasis of PDAC cells via regulation of localized translation in cell protrusions. Analysis of pancreatic xenograft tumors derived from control-RNAi S2-013 cells showed that cytoplasmic granular IGF2BP3 was mainly observed in the tumor penumbra, and ARF6 was strongly expressed near cell membranes in the penumbra. In contrast, pancreatic xenograft tumors derived from *IGF2BP3*-RNAi cells had less ARF6 at the cell membranes than did tumors derived from control-RNAi cells. These findings indicated that, for the xenograft tumors that derived from control-RNAi S2-013 cells, the expression of granular IGF2BP3 may have been interrelated with expression of membranous ARF6 in the penumbra. At the leading edges of invasive tumors in the penumbra regions, PDAC cells are endowed with increased migratory capacity and augmented invasiveness [25]. We showed that *IGF2BP3*-knockdown in primary pancreatic xenograft tumors completely blocked metastasis to liver and lung *in vivo*. Therefore, cell populations that overexpress ARF6 near cell membranes in the tumor penumbra may have a powerful advantage with regard to invasiveness and metastasis. Like *ARF6* and *ARHGEF4* transcripts, the other IGF2BP3-bound mRNAs were significantly associated with GO terms relevant to cell motility, invasiveness, and protrusions (Figure 4A). Therefore, the IGF2BP3-bound mRNAs that were preferentially translated in cell protrusions may have contributed to cell invasion and strongly modulate metastasis to lung and liver in cases of PDAC.

RNA-binding proteins (hnRNP K, hnRNP E1, and FUS/TLS) and ribosomal RNA are present in recently described structures called spreading initiation centers (SICs), which are similar to, but distinct from, the more mature focal adhesions [26]. Interfering with the function of the RNA-binding proteins localized in SICs results in increased cell spreading [26]. These previous findings and specifically the finding that RNA-binding proteins and ribosomal RNA colocalize in SICs raise the possibility that localized translation of selective mRNAs may play a role in cell spreading. However, de Hoog et al. also showed that β-actin and focal adhesion kinase (*FAK*) mRNAs were not present in SICs; notably, FAK is a widely expressed cytoplasmic protein tyrosine kinase located in integrin-mediated focal adhesions [27]. While the identities of mRNAs localized in SICs are still unknown, we used a next generation sequencer to identify a number of IGF2BP3-bound mRNAs that might be necessary for cell invasion and metastasis. Our studies present the first direct evidence for a role of IGF2BP3 in the local translation of target mRNAs in cell protrusions and consequently in the invasiveness and metastasis of PDAC cells. The data presented here indicated to us that inhibition of 1) IGF2BP3, 2) IGF2BP3-bound transcripts assembled in cell protrusions, 3) the local translational system in cell protrusions, or 4) some combination thereof may be effective for targeted molecular therapy because any such therapy would inhibit local translation of specific mRNAs in cell protrusions and consequently limit the invasiveness and metastasis of PDACs.

## METHODS AND MATERIALS

### Antibodies

Rabbit anti-IGF2BP3 (2037) and anti-ARHGEF4 (18267) antibodies were purchased from Human Protein Atlas (Stockholm, Sweden). Anti-G3BP monoclonal antibody (611126) was purchased from BD Transduction Laboratory (Palo Alto, CA). Polyclonal antibody against TIA-1 (1751) and monoclonal antibody against c-myc (40) were purchased from Santa Cruz Biotechnology (Santa Cruz, CA). Rabbit anti-ARF6 antibody (77581) was purchased from Abcam (Cambridge, MA).

### Cell culture and reagents

The human PDAC cell line S2-013, a subline of SUIT-2, was obtained from Dr. T. Iwamura (Miyazaki Medical College, Miyazaki, Japan) [15]. The human PDAC cell line PANC-1 was purchased from the American Type Culture Collection (Manassas, VA). All cells were grown in Dulbecco's modified Eagle's medium (DMEM; Gibco-BRL, Carlsbad, CA) supplemented with 10% heat-inactivated fetal calf serum (FCS) at 37°C in a humid atmosphere saturated with 5% CO_2_. To induce oxidative stress, plated cells were treated for 30 min with sodium arsenite (500 μM; Sigma-Aldrich, St. Louis, MO).

### Confocal immunofluorescence microscopy

Coverslips were coated with 10 μg/mL fibronectin (Sigma-Aldrich) for 1 h at room temperature. Cells were seeded on fibronectin-coated glass coverslips and incubated for 5 h; cells were then fixed with 4% paraformaldehyde, permeabilized with 0.1% Triton X-100, covered with blocking solution (3% BSA/PBS), and then incubated with the appropriate primary antibody for 1 h. Alexa488-, Alexa546-, Alexa594-, or Alexa647-conjugated secondary antibody (Molecular Probes, Carlsbad, CA) was used with or without rhodamine-conjugated phalloidin (Cytoskeleton, Denver, CO). In some experiments, a commercial antibody-labeling technology (Zenon; Life Technologies, Carlsbad, CA) was used according to the manufacturer's instructions to conjugate green or red fluorophores to primary antibodies. Each specimen was visualized using a Zeiss LSM 510 META microscope (Carl Zeiss, Gottingen, Germany).

### Generation of a S2-013 cell line that stably expressed siRNA

Exponentially growing GP2-293 packaging cells (Clontech, Mountain View, CA) were transiently infected with pGFP-V-RS vectors (OriGene Technologies, Rockville, MD) to generate replication-deficient lentivirus that carried a small interfering RNA (siRNA) expression cassette targeting either a scrambled negative control (TR30013), or *IGF2BP3* mRNA (TG312221). Upon transient transfection of the plasmids into the packaging cell line, replication-deficient viruses were obtained and used to infect S2-013 cells; infected S2-013 cells were transferred to flasks 48 h after infection and then grown in DMEM containing 0.5 μg/mL puromycin (Sigma-Aldrich) for 7 days to establish S2-013 cells that stably expressed the appropriate siRNA that targeted *IGF2BP3* mRNA. For each experiment, these cells were cultivated until they reached confluence and then for an additional 10 days; medium was refreshed every second day during cell cultivation. Cells were used only when suppression of IGF2BP3 had been validated via western blot analysis.

### IGF2BP3-rescue construct

Reverse transcription-PCR (RT-PCR) was used to amplify the entire coding sequence of the *IGF2BP3* cDNA. The resultant PCR product was subsequently inserted into a separate pCMV6-Entry vector (Origene) bearing a C-terminal myc-DDK-tag. X-tremeGENE HP DNA Transfection Reagent (Roche, Penzberg, Germany) was used to transiently transfect target cells with resultant the IGF2BP3-rescue construct.

### Wound-healing motility assay

For each wound-healing assay, a plastic pipette tip was used to cut cross-shaped wounds through a confluent cell monolayer. Several wound areas were marked for orientation and were then photographed using phase-contact microscopy. Marked wounds were repeatedly photographed. In any one experiment, the overall duration of wound healing varied from 1 to 8 h, and the degree of wound closure was quantified. The number of cells that had migrated into an initially cell-free wound area was determined and recorded.

### Trans-well motility assay

Cells (3.0 × 10^4^) were plated in the upper chamber of BD BioCoat Control Culture Inserts (24-well plates, 8-μm pore size; Becton Dickinson, San Jose, CA). Serum-free culture medium was added to each upper chamber, and medium containing 5% FCS was added to each bottom chamber. Cells were incubated on the membranes for 12 h. After a 12-h incubation, three independent visual fields were examined via microscopic observation to count the number of cells that had moved to the bottom chamber.

### Matrigel invasion assay

A two-chamber invasion assay was used to assess cell invasion (24-well plates, 8-μm pore size membrane coated with a layer of Matrigel extracellular matrix proteins; Becton Dickinson). Cells (4.0 × 10^4^) suspended in serum-free medium were seeded into the upper chamber and allowed to invade towards a 5% FCS chemoattractant in the lower chamber. After a 20-h incubation, three independent visual fields were examined via microscopic observation, and the number of cells that had moved to the bottom chamber was determined.

### Mice and orthotopic implantation of tumor cells

Pathogen-free female athymic nude mice (BALB/cSlc-*nu/nu*, 6 weeks of age) were purchased from Japan SLC, Inc (Shizuoka, Japan). Mice were treated in accordance with the Institutional Animal Care and Use Committee guidelines of Kochi University. Cells (8.0 × 10^5^) were surgically and orthotopically implanted into the pancreas of each mouse. Each mouse was sacrificed 42 days after the respective implantation; hematoxylin and eosin staining was then used to determine the presence or absence of tumor invasion into the retroperitoneum and of metastatic lesions in the lung and liver. Pancreatic tumors were excised, examined, and weighed.

### Immunoprecipitation

S2-013 cells were incubated on fibronectin for 5 h, lysed in lysis buffer [50 mM Tris (pH 7.4), 150 mM NaCl, 1 mM MgCl_2_, 0.5% NP-40, and protease inhibitor cocktail tablets (Roche)], and the resulting lysates were immunoprecipitated with 2 μg of anti-IGF2BP3 antibody or rabbit IgG isotype control antibody, and Dynabeads Protein G (Dynal, Oslo, Norway). To examine the interaction between endogenous IGF2BP3 and G3BP, immune complexes were analyzed on western blots.

### RNA immunoprecipitation (RIP), next-generation sequencing, and bioinformatics analysis

Cells were seeded onto fibronectin and incubated for 5 h. Cells were washed twice with PBS, and then lysed in NP2 buffer containing 50 mM Tris (pH 7.4), 150 mM NaCl, 1 mM MgCl_2_, 0.5% NP-40 and protease, and RNase inhibitors (Roche). Lysates were immunoprecipitated with Dynabeads Protein G (Dynal) and with anti-IGF2BP3 antibody or with rabbit IgG isotype control antibody for 2 h at 4ºC. Beads were pelleted on a magnetic rack (Dynal). The precipitated complexes were treated with proteinase K, extracted with phenol-chloroform, and nucleic acids were precipitated with ethanol. Precipitated nucleic acids were subsequently treated with DNase I (Promega, Madison, WI), and RNA was purified using the RNeasy kit (Qiagen, Valencia, CA) according to the manufacturer's instructions. RNA samples were processed by Hokkaido System Science (Sapporo, Japan) using an Illumina/Solexa instrument following standard procedures. For the bioinformatic analysis of the data obtained, Illumina/Solexa reads from the anti-IGF2BP3 RIP (11,203,904) and from the control (rabbit IgG isotype control antibody) (11,162,563) RIP were mapped to the mRNAs from RefSeq [28] using the LSKB Database (World Fusion Co., LTD., Tokyo, Japan). The correlation between the anti-IGF2BP3 sample and the isotype control sample can be seen in Figure S1. We used a measure, designated RPKM (reads per kilobase per million of mapped reads), to approximate the density of each mRNA within the anti-IGF2BP3 RIP [29]. We used the adjusted mean and standard deviation of the log_2_ (*RPKM)* value of each sample to normalize the RPKM estimates. We regarded transcripts with a log_2_ ratio (RPKM from IGF2BP3 sample / RPKM from isotype control sample) > 1.0 as transcripts that potentially bound IGF2BP3 (2,826 genes). Gene Ontology analyses were performed for genes represented by IGF2BP3-bound transcripts. Statistically significant biological process terms were obtained using PathwayStudio® [30] (Ariadne Genomics, Inc, Rockville, MD) by World Fusion Co., LTD.

### RNA immunoprecipitation and RT-PCR

Purified RNAs obtained via RIP were subjected to reverse transcription (RT) with StrataScript reverse transcriptase (Agilent, La Jolla, CA) and oligo d(T)_12-18_ primers. We prepared appropriate dilutions of each single-stranded cDNA for subsequent PCR amplification; *Ubiquitin C* mRNA was used as an internal quantitative control. The primer sequences used to amplify *ARF6* and *ARHGEF4* are available on request.

### Immunofluorescence with RNA fluorescence *in situ* hybridization

The QuantiGene ViewRNA plate-based assay kit (Panomics; Santa Clara, CA) was used according to the manufacturer's recommendations with some modifications [31] to perform fluorescence *in situ* hybridization to target RNAs. Fibronectin-stimulated S2-013 cells were fixed in 8% formaldehyde, dehydrated in ethanol (50%-70%-100%), and held at 4°C overnight. Cells were then rehydrated, permeabilized, and hybridized as recommended. The RNA targets were *ARF6* or *ARHGEF4* (Panomics), and the reference RNA was *ubiquitin C* (*UBC*) (Panomics). After *in situ* hybridization, sections were washed in PBS, blocked for 1 h with blocking buffer (4% goat serum in PBS), and incubated for 3 h at room temperature with anti-IGF2BP3 antibody in blocking buffer. Secondary antibodies in blocking buffer were applied to the samples for 30 min at room temperature, nuclei were stained for 3 min with DAPI, and samples were mounted in Aqua Polymount (Polysciences, Warrington, PA). Confocal fluorescence images were captured with a Zeiss LSM 510 META microscope.

### Immunohistochemical staining

Paraffin-embedded tissue sections from control or *IGF2BP3*-RNAi S2-013 primary pancreatic tumors in mice were deparaffinized and autoclaved at 108°C for 15 min. After endogenous peroxidase activity was quenched by incubation for 30 min in 0.33% hydrogen peroxide diluted in methanol, the sections were incubated with fetal bovine serum for blocking. Sections were then incubated with anti-IGF2BP3 or anti-ARF6 antibody at room temperature for 1 h and washed with PBS. Immunodetection was performed with peroxidase-labeled anti-rabbit immunoglobulin (Dako Cytomation, Carpinteria, CA). Finally, the reactants were developed with 3,3′-diaminobenzidine (Dako), and the sections were counterstained with hematoxylin.

### siRNA treatment

Mixtures of four different siRNA oligonucleotides that targeted *ARF6* or *ARHGEF4* were purchased from Qiagen (FlexiTube GeneSolution: GS382 and GS50649, respectively), and mixtures of four different scrambled negative control siRNA oligonucleotides were purchased from Santa Cruz Biotechnology (37007). To examine the effect of a siRNA on *ARF6* or *ARHGEF4* expression, S2-013 or PANC-1 cells that expressed ARF6 and ARHGEF4 were plated in six-well plates. After 20 h, the cells were transfected with 80 pmols of siRNA in siRNA transfection reagent (Qiagen) following the manufacturer's instructions. After 48 h further incubation, the cells were used for immunocytochemistry, trans-well motility assays, or Matrigel invasion assays.

### Statistical analysis

GraphPad Prism software (version 6.0, GraphPad Software, Inc, La Jolla, CA) was used for all statistical analyses. The significance of differences between groups was determined using the two-tailed Student's *t*-test or Fisher's exact test, as appropriate. For all analyses, *p* < 0.05 was considered statistically significant.

## SUPPLEMENTARY MATERIAL FIGURE AND TABLES



## Abbreviations

PDAC: pancreatic ductal adenocarcinoma
SG: stress granule
siRNA: small interfering RNA
RNAi: RNA interference
RT-PCR: reverse transcription-PCR
IP: immunoprecipitation
RIP: RNA immunoprecipitation
SIC: spreading initiation center.
