# Supplementary material for: IGF2BP3-mediated translation in cell protrusions promotes cell invasiveness and metastasis of pancreatic cancer
Source: Oncotarget. 2014 Jul 25;5(16):6832–45. doi: 10.18632/oncotarget.2257 (PMC4196166; doi:10.18632/oncotarget.2257)
Supplement: Supplementary file 1 [file oncotarget-05-6832-s001.pdf]

IGF2BP3-mediated translation in cell protrusions promotes cell invasiveness and metastasis of pancreatic cancer

Supplementary Material

Figure S1

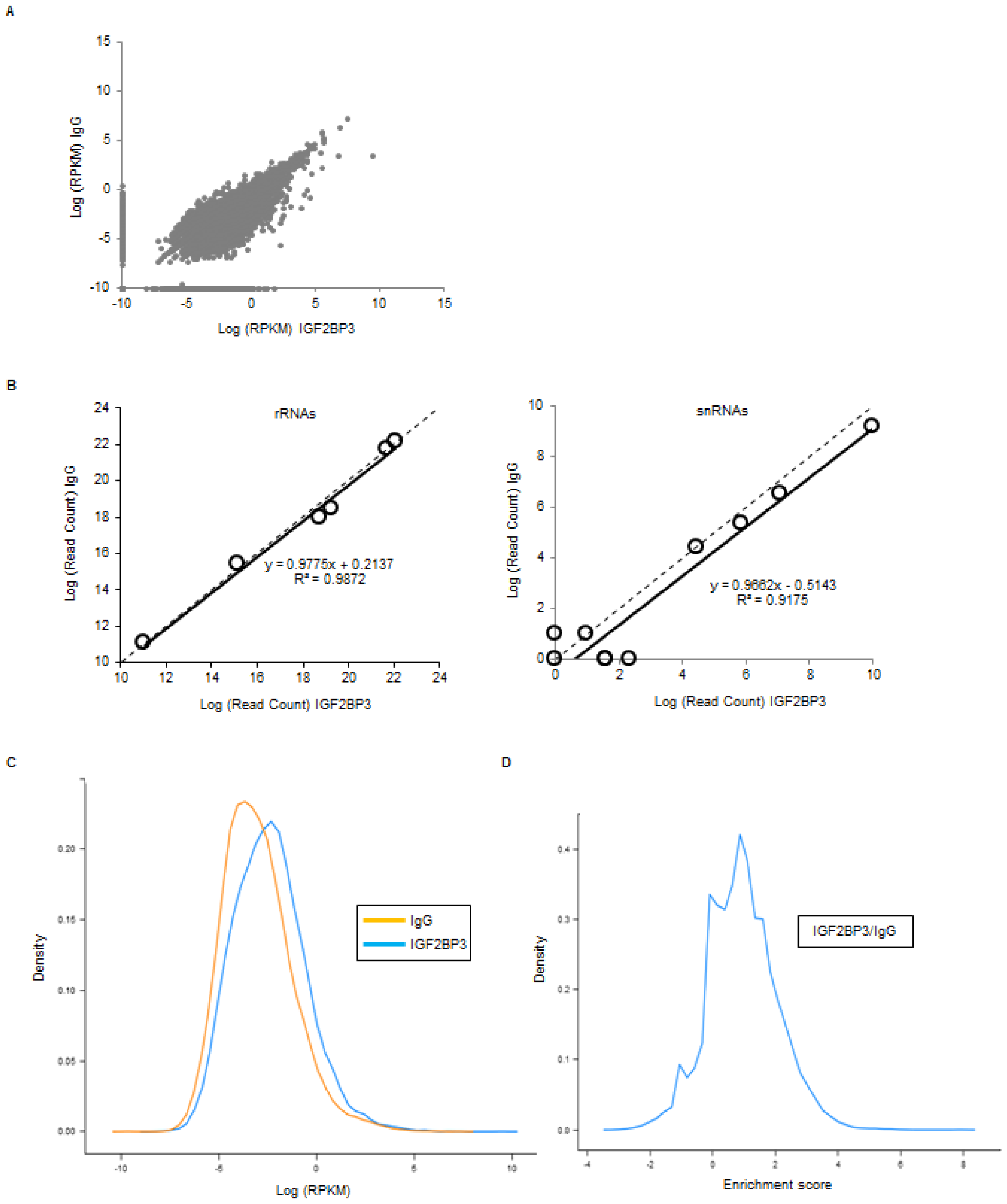

Figure S1: Ultrasequencing analysis. (A) Scatter-plot of the read-densities for the mRNAs in IGF2BP3 IP samples or in control (IgG) IP samples. Read-densities are calculated using the *RPKM* (reads per kilobase per million of mapped reads) measure. (B) Linear regression of the read counts in ribosomal RNAs (rRNAs) and small nuclear RNAs (snRNAs) between two samples, IGF2BP3 IP and control (IgG) IP. The dashed line represents the regression line. The dotted line represents the x = y line. The correlation factors are R^2 = 0.9872 for rRNAs and R^2 = 0.9175 for snRNAs. (C) Distributions of the log2(*RPKM*) values for IGF2BP3 IP reads and control IP reads. (D) Distribution of the enrichment scores for IGF2BP3 IP over control IP. Here we only considered mRNAs for which we had at least 1 read in the IGF2BP3 IP and control IP.

Table S1: mRNAs that co-immunoprecipitate with IGF2BP3

Gene	Entrez Gene ID	Log ratio RPKM(IGF2BP3)/RPKM(control IgG)
psiTPTE22	387590	5.695564097
LOC100188947	100188947	5.606738317
C13orf23	80209	5.575312331
UCA1	652995	5.424214379
MLL5	55904	5.241795493
CDR1	1038	5.217418799
SNORA12	677800	5.082787732
WAC	51322	4.964398632
MUC16	94025	4.761817143
TAB2	23118	4.721919446
LOC349408	349408	4.691307058
ATN1	1822	4.607477888
NFAT5	10725	4.581953751
SNORD22	9304	4.568640195
R3HDM1	23518	4.417948749
SH3BGRL3	83442	4.383975217
RNU12	267010	4.326249701
HHLA3	11147	4.317691102
ELK3	2004	4.191215645
NCOA6	23054	4.186659017
PLBD2	196463	4.159437346
DYX1C1	161582	4.152994605
QSER1	79832	4.148934105
RAB8A	4218	4.13923066
ABO	28	4.097950448
POM121	9883	4.022040797
OAF	220323	3.968840508
SNORA18	677805	3.916476644
BCAM	4059	3.843439382
SLC25A23	79085	3.834118153
MBD6	114785	3.819156895
BRD4	23476	3.805449977
LDOC1	23641	3.787641414
GSK3A	2931	3.778208576
CNOT4	4850	3.713306569
EP300	2033	3.645451405
LSM12	124801	3.641743382
EGR1	1958	3.641521102
ATP11A	23250	3.613280771
FOXJ3	22887	3.607626221
RBPMS	11030	3.584962501
ASAP1	50807	3.563759228
KIAA0114	57291	3.54225805
HIST2H2AB	317772	3.478971805
SPC25	57405	3.469885976
C17orf63	55731	3.428946345
TRERF1	55809	3.427734812
ARHGAP17	55114	3.427606173

Gene	Entrez Gene ID	Log ratio RPKM(IGF2BP3)/RPKM(control IgG)
TOMM5	401505	3.420886575
PACS1	55690	3.404518746
PATL1	219988	3.40152957
SLC29A2	3177	3.365972428
HGSNAT	138050	3.340562269
FOSL1	8061	3.292711933
TRIM14	9830	3.292289884
CDC42EP2	10435	3.292151514
FADS1	3992	3.279025492
UPK2	7379	3.264836648
ZFAND3	60685	3.258971109
FGFRL1	53834	3.258835834
MAML2	84441	3.254896336
FOXP4	116113	3.247864183
FAM86C	55199	3.237410753
LOC100129034	100129034	3.225278774
CYP2S1	29785	3.217011084
ISG20L2	81875	3.206486944
RNF187	149603	3.205861657
HMGA1	3159	3.202455364
GPATCH8	23131	3.194244681
SH2B3	10019	3.174174474
PBX2	5089	3.168962884
MGRN1	23295	3.162118959
MPV17	4358	3.157690514
NME4	4833	3.139025508
TJP1	7082	3.134316655
MCFD2	90411	3.128326192
ZMIZ1	57178	3.118921816
MYD88	4615	3.114700172
ZNF384	171017	3.106722214
UNC93B1	81622	3.105845667
FAM65A	79567	3.104887035
SSH3	54961	3.098091577
C15orf23	90417	3.086474065
ICMT	23463	3.085594615
CD68	968	3.08529578
CAPNS1	826	3.084428781
ARID1A	8289	3.083019586
FAM127B	26071	3.080145979
SNHG6	641638	3.078097423
RPRD2	23248	3.077656334
SLC25A22	79751	3.07302493
TAB3	257397	3.065227623
LMLN	89782	3.061948965
SMURF2	64750	3.057084048
ITM2B	9445	3.056473736
GORASP2	26003	3.056413309
NMNAT2	23057	3.056064622

Gene	Entrez Gene ID	Log ratio RPKM(IGF2BP3)/RPKM(control IgG)
SMG7	9887	3.04454332
R3HDM2	22864	3.039163056
KCNN2	3781	3.036854953
WNK1	65125	3.023995481
PABPC1	26986	3.020460931
KIAA0494	9813	3.01913186
SNORA54	677833	3.018100008
NDUFB2	4708	3.015247774
ANKRD17	26057	3.014060355
LOC442293	442293	3.009764917
POGZ	23126	3.001982343
UBAP2	55833	2.988828071
C12orf32	83695	2.987866549
SNORA27	619499	2.983495294
PGRMC1	10857	2.969481962
TBCA	6902	2.96936012
SERINC2	347735	2.969265181
PLAC8	51316	2.969112145
TFG	10342	2.956614288
BPTF	2186	2.952653272
SSSCA1	10534	2.945045797
FHL1	2273	2.932230183
SNORA45	677826	2.927327605
MRPL4	51073	2.916285546
ANKRD36BP1	84832	2.916285546
SDF4	51150	2.907845075
SNORA21	619505	2.9053509
POLR2A	5430	2.904145219
BANF1	8815	2.900804224
NOP10	55505	2.897240426
FNDC3B	64778	2.889649838
VAMP2	6844	2.8871353
FLJ42627	645644	2.885769574
SNORA63	6043	2.88381629
NPAS2	4862	2.883349338
EIF1AD	84285	2.875747213
SNORA71B	26776	2.873222616
CHCHD10	400916	2.871055075
TIPARP	25976	2.864532845
SNORA67	26781	2.862748926
POGK	57645	2.856587375
NPIP	9284	2.855591108
RERE	473	2.853447014
MALL	7851	2.851901361
NFIC	4782	2.851817708
CABLES2	81928	2.829443681
CORO2B	10391	2.81747727
ZFP36L1	677	2.816927601
HIVEP3	59269	2.80981616

Gene	Entrez Gene ID	Log ratio RPKM(IGF2BP3)/RPKM(control IgG)
STK39	27347	2.807767062
CCDC6	8030	2.805377026
BRPF3	27154	2.802468567
ALKBH5	54890	2.801158656
PAICS	10606	2.800973271
ASXL2	55252	2.799424894
UXT	8409	2.793271654
MLL	4297	2.789997352
LOC92249	92249	2.787850334
NFATC3	4775	2.785968967
MRPS21	54460	2.776955325
TRIOBP	11078	2.776817752
ZFP91	80829	2.776760316
PORCN	64840	2.776680893
ELK4	2005	2.776608299
CTNNBL1	56259	2.776465049
C11orf41	25758	2.776404832
RAD23B	5887	2.776164487
CCDC124	115098	2.775685965
IFITM2	10581	2.772941338
COPZ1	22818	2.771674334
CA12	771	2.771251752
HNF1B	6928	2.760220946
EGFR	1956	2.760059149
IMPDH1	3614	2.752534526
ZNF142	7701	2.751677946
PHLDA1	22822	2.746627733
CCNK	8812	2.746425923
RAPH1	65059	2.7322692
TICAM1	148022	2.730748629
LRRC8A	56262	2.730313886
GLI2	2736	2.728138203
PRR14	78994	2.720278465
ABLIM1	3983	2.716538083
RAB7A	7879	2.714369551
MED15	51586	2.71117765
LRCH4	4034	2.709511214
NHSL1	57224	2.709511214
RELA	5970	2.706514233
EMP2	2013	2.706170111
MUC5B	727897	2.700439718
TPP1	1200	2.697551439
KCMF1	56888	2.69265037
SLC9A3R1	9368	2.691985847
FAM127A	8933	2.689299161
MCCC2	64087	2.682184308
WBP5	51186	2.681898983
CDC42SE1	56882	2.669820568
RAB6A	5870	2.669799743

Gene	Entrez Gene ID	Log ratio RPKM(IGF2BP3)/RPKM(control IgG)
SHFM1	7979	2.66970077
UBP1	7342	2.669337011
TFAP2A	7020	2.669213837
SP1	6667	2.66917897
SGPP2	130367	2.669122948
WASL	8976	2.658782734
HMG20B	10362	2.655122811
SAP130	79595	2.65511384
UBXN2B	137886	2.653747943
MOBKL2A	126308	2.652142272
SF3A2	8175	2.647310407
ZNF462	58499	2.644317778
DBNDD1	79007	2.642239466
BAD	572	2.625270489
SRCAP	10847	2.620241336
PPP1R11	6992	2.613304838
CRTC2	200186	2.612598578
BCL7B	9275	2.612454379
GRIN2D	2906	2.600983311
MBNL1	4154	2.588408156
ATP6V1D	51382	2.578697238
PTPN23	25930	2.576122013
LCLAT1	253558	2.574101508
SEC24B	10427	2.567058626
TIMM10	26519	2.564134243
ZMIZ2	83637	2.561766521
PIP4K2C	79837	2.558426219
PTPLB	201562	2.554834396
SCARNA9	619383	2.554546395
CDA	978	2.554358039
GTF3A	2971	2.55429245
TEAD4	7004	2.554290805
TMEM208	29100	2.554253603
TXNL1	9352	2.554190152
BRI3	25798	2.554183336
PPPDE2	27351	2.554015932
RCOR1	23186	2.554006432
UBXN6	80700	2.553605382
RRAS	6237	2.553521813
PDCD5	9141	2.552990111
MRPL50	54534	2.549915554
TOMM34	10953	2.545721311
SLC25A10	1468	2.540523296
DPM2	8818	2.536301619
NUP214	8021	2.536176955
ARHGAP21	57584	2.531526144
COPS8	10920	2.521553331
MLF2	8079	2.518994854
ATXN2L	11273	2.517700283

Gene	Entrez Gene ID	Log ratio RPKM(IGF2BP3)/RPKM(control IgG)
SUN1	23353	2.517023672
PTPN12	5782	2.514716007
C20orf111	51526	2.513238062
IFI35	3430	2.512985335
CIZ1	25792	2.511212646
IMP4	92856	2.510961919
MED21	9412	2.506906555
HIPK3	10114	2.504111864
FAM89B	23625	2.500292177
DLGAP4	22839	2.49940337
C20orf11	54994	2.493134922
CNBP	7555	2.493094783
BET1L	51272	2.4923183
POU2F1	5451	2.488257913
EPB49	2039	2.487891839
C11orf30	56946	2.486971744
HNRNPUL1	11100	2.48512614
C16orf13	84326	2.484911487
IGF2	3481	2.484395963
RASA1	5921	2.482590175
PHC3	80012	2.481040556
RAD23A	5886	2.47193053
EHD2	30846	2.471333467
ABHD2	11057	2.471102839
TIMD4	91937	2.462324863
PLEKHG2	64857	2.460218329
TUG1	55000	2.452331857
KLF7	8609	2.45206823
POM121C	100101267	2.451448711
LOC613037	613037	2.451013243
WIPF2	147179	2.446974269
APOOL	139322	2.445991491
TCF7L2	6934	2.440248062
EVC	2121	2.439357178
NR2F2	7026	2.438475704
CDCP1	64866	2.433892105
RNF6	6049	2.431890348
ITPRIP	85450	2.428946345
ATXN2	6311	2.428922627
UFC1	51506	2.428775203
PTP4A2	8073	2.428734074
SCMH1	22955	2.428482379
KCTD13	253980	2.428462225
C19orf50	79036	2.428163528
PAQR5	54852	2.427606173
LOC646214	646214	2.427432726
CHD2	1106	2.421425296
SCARNA16	677781	2.421425296
BCAP29	55973	2.419808529

Gene	Entrez Gene ID	Log ratio RPKM(IGF2BP3)/RPKM(control IgG)
TEX261	113419	2.413864833
TRAPPC1	58485	2.4120491
IWS1	55677	2.410069692
RBM33	155435	2.408168371
CREBBP	1387	2.407310945
SRRM2	23524	2.40355421
PPP1R15B	84919	2.403540418
P2RY2	5029	2.402562097
LYPD3	27076	2.394239735
ANO1	55107	2.389265198
DGUOK	1716	2.388189537
ZDHHC12	84885	2.38708677
SLC41A1	254428	2.384541843
PAPOLA	10914	2.384437296
GNA11	2767	2.384390903
SPRYD4	283377	2.381837084
KIAA1949	170954	2.373947403
ECHDC1	55862	2.373787394
RAB5B	5869	2.37159098
AGFG1	3267	2.371280054
EPAS1	2034	2.370930642
HIPK2	28996	2.369925872
VEZF1	7716	2.367930141
OGFRL1	79627	2.367371066
CD276	80381	2.365145694
TGOLN2	10618	2.364060724
IDS	3423	2.363731783
ZDHHC8	29801	2.361588182
PLAGL2	5326	2.361456459
ARL1	400	2.36093729
HOXB7	3217	2.360645202
C12orf35	55196	2.358114898
SH3GL1	6455	2.356123015
KDM3B	51780	2.355862006
KIAA2026	158358	2.355862006
ANXA11	311	2.355575101
PPPDE1	51029	2.355016264
HSPA12A	259217	2.353284519
RBM22	55696	2.350214338
TRIM26	7726	2.347782133
EFNB2	1948	2.344260395
RFFL	117584	2.344260395
CYB5R3	1727	2.342847913
TEAD1	7003	2.342554745
CDK2AP2	10263	2.340847077
B4GALT1	2683	2.338098438
CITED2	10370	2.33714018
NAT15	79903	2.335265894
EHMT2	10919	2.328836464

Gene	Entrez Gene ID	Log ratio RPKM(IGF2BP3)/RPKM(control IgG)
KIAA1267	284058	2.327974725
DNAJC17	55192	2.327112471
ROD1	9991	2.32704858
CYB561	1534	2.325098538
CDK6	1021	2.324248473
PHC1	1911	2.323946455
CMTM7	112616	2.318908231
PRDM2	7799	2.317882883
TGFB1	7040	2.317593505
ERBB2IP	55914	2.317405163
SNORA74A	26821	2.316725022
EIF2AK1	27102	2.313638123
PEX10	5192	2.308302621
TRIM29	23650	2.308302621
ELL2	22936	2.30626224
GLIS3	169792	2.302465287
CMIP	80790	2.294040783
TRAM2	9697	2.291607644
TAX1BP3	30851	2.291483688
RAGE	5891	2.291466991
CLDN1	9076	2.291462814
ZNHIT3	9326	2.291347494
LOC652276	652276	2.291341607
MLX	6945	2.291231298
RNF126	55658	2.291231298
LENG8	114823	2.291219197
RNU11	26824	2.291183983
CINP	51550	2.291141969
ELOF1	84337	2.29113036
PCBD1	5092	2.291093331
COX6A1	1337	2.291015364
YLPM1	56252	2.291008133
LHPP	64077	2.290915912
ARF5	381	2.290828891
PTPN14	5784	2.29080403
MAP7	9053	2.290790611
FLOT1	10211	2.290771688
VKORC1L1	154807	2.290760077
RPF2	84154	2.290696771
CIRBP	1153	2.290561752
MCRS1	10445	2.290530895
PSENEN	55851	2.290491436
ZNF532	55205	2.290486376
C16orf74	404550	2.290399526
WDR45L	56270	2.290338573
RARA	5914	2.290129465
NDUFB6	4712	2.286585483
CCDC90A	63933	2.283625501
ZNF488	118738	2.277389699

Gene	Entrez Gene ID	Log ratio RPKM(IGF2BP3)/RPKM(control IgG)
CKLF	51192	2.275896786
PAXIP1	22976	2.272918995
ARHGEF35	445328	2.271724464
SMARCD1	6602	2.270229907
SCOC	60592	2.267236142
UBE2E3	10477	2.264985312
VPS24	51652	2.264836648
CNOT1	23019	2.264803696
ZBTB20	26137	2.259423152
CCNC	892	2.25761413
ATF7	11016	2.253686783
ITGA2	3673	2.251873202
ZNF385A	25946	2.251264629
ARHGEF5	7984	2.248804052
TSPAN14	81619	2.24842661
C11orf24	53838	2.246408087
ARPP19	10776	2.242461413
ADIPOR2	79602	2.242051953
CDK16	5127	2.2410081
SPRED2	200734	2.236339539
SEPN1	57190	2.236033332
H1F0	3005	2.234501321
LHX1	3975	2.232523102
ELF4	2000	2.232142073
PLEKHB2	55041	2.231856167
TCERG1	10915	2.231824835
PGM5P2	595135	2.231460002
UBE2N	7334	2.229280279
GNG4	2786	2.228356954
SLCO3A1	28232	2.227741076
AKT3	10000	2.226816765
SNX8	29886	2.225891862
MED13L	23389	2.22527493
PRRC1	133619	2.22527493
FOXK2	3607	2.223456929
NPIPL3	23117	2.220639513
ZNF460	10794	2.220329955
TFAP4	7023	2.22002033
SNX12	29934	2.219091058
PRR11	55771	2.217451451
SIGMAR1	10280	2.216927211
ZFP36	7538	2.216818577
CCNI	10983	2.216795977
DAP	1611	2.216695691
HIST1H2BB	3018	2.212569339
RBM4B	83759	2.211635253
CRTC3	64784	2.210388864
CTDSP1	58190	2.206500711
C6orf132	647024	2.206314381

Gene	Entrez Gene ID	Log ratio RPKM(IGF2BP3)/RPKM(control IgG)
FOXRED2	80020	2.205079666
BACE1	23621	2.203514411
ZFHX3	463	2.202260984
MARCH7	64844	2.201320198
PRMT5	10419	2.200378798
PI4KB	5298	2.199436784
DVL3	1857	2.199353299
FKBP10	60681	2.19200635
MANEAL	149175	2.191878443
CHP	11261	2.191858927
DPF2	5977	2.19175592
RREB1	6239	2.191479722
TMEM212	389177	2.191411345
AUP1	550	2.191036564
VPS37B	79720	2.190942783
ARF6	382	2.187451054
ARIH1	25820	2.187451054
SERPINB9	5272	2.183645305
SAMD4B	55095	2.181762237
ARHGEF12	23365	2.181705485
TWF1	5756	2.179239678
ELOVL5	60481	2.177897481
ZBTB2	57621	2.176960992
ESYT2	57488	2.175364914
SOX9	6662	2.174313848
ONECUT2	9480	2.173767068
RAB3B	5865	2.17280751
POLR3H	171568	2.171236185
C15orf39	56905	2.171039697
CBX5	23468	2.170830364
CASC4	113201	2.17076152
PTPN1	5770	2.170759653
GLO1	2739	2.170568631
GMEB2	26205	2.168962884
BIRC3	330	2.164142649
KCTD9	54793	2.164142649
DCBLD2	131566	2.163454263
PLRG1	5356	2.161565256
MYEOV2	150678	2.159952045
MLH1	4292	2.158660175
FAM189B	10712	2.158660175
TRIM41	90933	2.157690514
SNHG1	23642	2.157367148
ARHGDIB	397	2.153294831
FLYWCH2	114984	2.152795565
DIABLO	56616	2.151534141
FBRS	64319	2.151083887
SPEN	23013	2.150087632
ADRA1B	147	2.144372734

Gene	Entrez Gene ID	Log ratio RPKM(IGF2BP3)/RPKM(control IgG)
TMED1	11018	2.141432791
EIF4A2	1974	2.139570335
FTSJ2	29960	2.139511164
SETD7	80854	2.139469495
PTPLAD1	51495	2.13922368
CLPB	81570	2.13920712
DAZAP1	26528	2.139189408
ACO2	50	2.139179487
MAPK13	5603	2.139143537
TOR1AIP2	163590	2.139108599
RHOD	29984	2.138851616
B4GALT4	8702	2.138809393
MRPL24	79590	2.138758587
TXNDC12	51060	2.138685071
TMEM131	23505	2.138625433
CDC45	8318	2.138486537
ZDHHC5	25921	2.138481954
NCBP2	22916	2.138409536
SMOX	54498	2.138350692
MEMO1	7795	2.13834401
DYRK1A	1859	2.136847604
SPATS2L	26010	2.136737516
FIP1L1	81608	2.128048361
ERGIC1	57222	2.12654216
SEMA6A	57556	2.123666196
ACTR3	10096	2.121147899
GPR126	57211	2.120842397
GMFB	2764	2.12035194
HIST1H2AK	8330	2.118359726
CSNK1E	1454	2.106928573
MAL2	114569	2.106544474
RFC4	5984	2.105678078
GLIS2	84662	2.10534284
MRPL21	219927	2.104672131
LYPLA2	11313	2.10400111
AVPI1	60370	2.102658131
TCF7	6932	2.101313901
MGC72080	389538	2.098621677
ETF1	2107	2.098556136
IMPAD1	54928	2.096261853
EIF1AX	1964	2.095586908
SRPK1	6732	2.09428473
TMEM97	27346	2.09423607
TTC1	7265	2.088141597
BTBD7	55727	2.086444109
CYP2B6	1555	2.085424656
CSNK2A1	1457	2.085421024
SGK196	84197	2.084632749
MET	4233	2.084469936

Gene	Entrez Gene ID	Log ratio RPKM(IGF2BP3)/RPKM(control IgG)
TMEM123	114908	2.084428781
VOPP1	81552	2.084352702
C5orf43	643155	2.084328206
SMYD5	10322	2.084178633
FAM100A	124402	2.080998686
ZNF828	283489	2.077242999
ARHGAP32	9743	2.073699431
GNA12	2768	2.073550251
PCGF2	7703	2.07347751
DCTN5	84516	2.069702167
ADNP	23394	2.069075996
CRTAP	10491	2.069014678
WBSCR16	81554	2.068326861
TOR1AIP1	26092	2.065916918
RAB11A	8766	2.058662839
PNKD	25953	2.056930288
SEPT11	55752	2.056924139
PSMA7	5688	2.056730989
FAM120A	23196	2.05664431
WBP2	23558	2.056523962
TMED2	10959	2.056489698
UBE2G2	7327	2.056335792
GRINA	2907	2.056261033
SRGAP2	23380	2.056111063
PPP1R13L	10848	2.056062966
USO1	8615	2.055853235
WDTC1	23038	2.053806444
RBBP6	5930	2.051372102
SLC9A1	6548	2.050327554
FAM167A	83648	2.049282249
DAG1	1605	2.047025335
AKAP13	11214	2.046936314
SERTAD2	9792	2.046826728
PODXL	5420	2.046722744
ALG3	10195	2.045842728
F5	2153	2.044394119
SIRPA	140885	2.043694461
MAP3K1	4214	2.040541794
SNF8	11267	2.040541794
CHERP	10523	2.039496995
ATG16L1	55054	2.039297625
CBARA1	10367	2.038787331
CREB5	9586	2.037030731
MRPS9	64965	2.037030731
EIF4E	1977	2.034215715
ABCC1	4363	2.030825994
TBC1D8	11138	2.030689204
ATOX1	475	2.029982866
TSPAN18	90139	2.028461328

Gene	Entrez Gene ID	Log ratio RPKM(IGF2BP3)/RPKM(control IgG)
WIZ	58525	2.028260381
GIT1	28964	2.028154234
TLK1	9874	2.028144502
TFDP1	7027	2.027882026
NDRG3	57446	2.027861775
POLR2E	5434	2.027799743
LOC643406	643406	2.025802819
UBE4A	9354	2.025383221
IL4R	3566	2.024783101
AFF4	27125	2.020413303
MAMLD1	10046	2.020050412
UNC5A	90249	2.019692601
HSP90AB2P	391634	2.018424957
KLF5	688	2.018132005
SSR1	6745	2.015782997
ZBTB38	253461	2.015782997
CD2AP	23607	2.013824046
SOX7	83595	2.010421805
SNX33	257364	2.003242417
UCK2	7371	2.001802243
FOXM1	2305	2.001052166
MED13	9969	2
NCOR1	9611	1.996760656
BNIP3L	665	1.99494171
MED1	5469	1.99442456
MRPS2	51116	1.99095486
MIER2	54531	1.988775562
ZRSR2	8233	1.98622888
RASAL2	9462	1.98550043
RAF1	5894	1.984771612
CLOCK	9575	1.982217846
PRAF2	11230	1.982217846
CD74	972	1.980767415
ZNF609	23060	1.975630186
EMB	133418	1.975263322
MBNL2	10150	1.972325042
TAF10	6881	1.971957335
LOC728643	728643	1.971737527
ZNF238	10472	1.969830779
C10orf2	56652	1.969749128
MRPS12	6183	1.969743505
DEF8	54849	1.969599114
NDUFB10	4716	1.969552789
RSU1	6251	1.96955093
BCL3	602	1.969532707
TNPO3	23534	1.969515692
PTTG1IP	754	1.96949081
HN1L	90861	1.969483573
ITGB3BP	23421	1.969467297

Gene	Entrez Gene ID	Log ratio RPKM(IGF2BP3)/RPKM(control IgG)
DDAH1	23576	1.96945808
COX7A2	1347	1.969457684
C20orf4	25980	1.969426756
UBFD1	56061	1.969403363
SMS	6611	1.969388907
CCDC97	90324	1.969375957
TMEM219	124446	1.969325971
NDUFAF3	25915	1.969303203
LEPREL1	55214	1.969279857
VPS26B	112936	1.969272467
DUSP5	1847	1.969245461
C11orf57	55216	1.969242604
AP1S1	1174	1.969137795
FDFT1	2222	1.969097384
WNT7A	7476	1.969006104
SHISA9	729993	1.968997799
RNF181	51255	1.968978618
EIF4E2	9470	1.968957578
SCARNA22	677770	1.968915802
DNAJC9	23234	1.968912322
CIR1	9541	1.968897247
HSBP1	3281	1.968856617
KLF3	51274	1.96884341
CDC27	996	1.968831657
EIF2AK2	5610	1.968786243
YTHDF1	54915	1.968777073
CRIM1	51232	1.968763501
RAB1B	81876	1.96870098
FKBP1A	2280	1.968659106
HIST1H2BO	8348	1.968640374
PLEK2	26499	1.968635829
ASAP2	8853	1.968629143
MOBKL3	25843	1.968614052
PDCL3	79031	1.968426551
KIAA0415	9907	1.96829114
LPCAT3	10162	1.965691949
TSG101	7251	1.964583463
SAP18	10284	1.963104155
COG4	25839	1.958285901
LARP1B	55132	1.956873193
MGLL	11343	1.956327524
TNS4	84951	1.953614946
ANO6	196527	1.952500854
PTPN18	26469	1.952333566
CAPRIN2	65981	1.951117794
C18orf10	25941	1.948600847
AMD1	262	1.946356572
TP53BP2	7159	1.945607703
PARVA	55742	1.944858446

Gene	Entrez Gene ID	Log ratio RPKM(IGF2BP3)/RPKM(control IgG)
LONP1	9361	1.942983598
MAP2K7	5609	1.942232976
SUOX	6821	1.941857519
SCARA3	51435	1.941857519
PPME1	51400	1.941223235
RNF44	22838	1.94073056
SAMD1	90378	1.94073056
PDZD8	118987	1.940653088
OSBPL9	114883	1.94057025
ZFR	51663	1.938720496
C8orf59	401466	1.93696766
ARHGAP29	9411	1.935836874
MAK16	84549	1.935754574
C14orf43	91748	1.935723978
YWHAQ	10971	1.935698516
PIP4K2B	8396	1.935459748
HRAS	3265	1.934327778
KIAA1310	55683	1.934327778
PPP1R13B	23368	1.932817103
PRKCA	5578	1.932264355
KIAA0947	23379	1.928753155
CAPZA1	829	1.928585667
SLC1A1	6505	1.928197304
DDX55	57696	1.925619712
YRDC	79693	1.925619712
KIAA1671	85379	1.925619712
MAPK1	5594	1.924585705
C21orf59	56683	1.924479992
NPNT	255743	1.92330491
SHISA5	51246	1.923169415
FAM133B	257415	1.921436331
CCDC137	339230	1.9202933
MLL3	58508	1.919530776
UBAP2L	9898	1.916746204
TMED10	10972	1.916254435
TLE3	7090	1.912266628
MLL2	8085	1.909736037
SNW1	22938	1.908812908
MOCS2	4338	1.907659828
SEC23IP	11196	1.907424828
WDR13	64743	1.907275263
RWDD1	51389	1.906890596
CTTNBP2NL	55917	1.906890596
ITPRIPL2	162073	1.906120953
SLC25A37	51312	1.904580435
CRKL	1399	1.904195048
SRC	6714	1.903423966
TMEM214	54867	1.902942002
PCBP2	5094	1.902891438

Gene	Entrez Gene ID	Log ratio RPKM(IGF2BP3)/RPKM(control IgG)
BMP2K	55589	1.902652471
CLPTM1L	81037	1.897240426
FAM122B	159090	1.897060314
PRKAA1	5562	1.896853073
KIAA1191	57179	1.896757942
ZNF80	7634	1.896078055
RNF157	114804	1.895108884
BICC1	80114	1.895055459
MED29	55588	1.89498755
TPD52	7163	1.894723487
SOS1	6654	1.893362211
ANXA3	306	1.888304895
TRIB1	10221	1.886628985
SDC3	9672	1.886355044
SMG1	23049	1.886260304
LZTR1	8216	1.885964757
TMEM111	55831	1.885183866
OSTC	58505	1.884011738
SPCS3	60559	1.882447416
NKTR	4820	1.881664619
NUP153	9972	1.879705766
DMKN	93099	1.878974903
KLHL36	79786	1.878529177
RCL1	10171	1.87774425
DDX52	11056	1.876409039
TBC1D16	125058	1.876251712
DNAJB6	10049	1.875873298
ERO1L	30001	1.875853509
UGCG	7357	1.875780063
SEPT8	23176	1.875780063
RNF26	79102	1.87563486
RALB	5899	1.875631878
MPG	4350	1.874600266
ZNF703	80139	1.874600266
EPS15	2060	1.873419503
FOXO3	2309	1.873419503
RALBP1	10928	1.873025701
CHD7	55636	1.869976697
ARHGAP27	201176	1.867658181
ARID2	196528	1.867501151
IFI16	3428	1.8667102
LOC100132247	100132247	1.866314561
WHSC2	7469	1.865126995
HNF4A	3172	1.863542051
TAOK3	51347	1.863145543
STARD7	56910	1.862359721
SETD5	55209	1.86235828
YTHDF3	253943	1.861896533
CDC42EP1	11135	1.861730976

Gene	Entrez Gene ID	Log ratio RPKM(IGF2BP3)/RPKM(control IgG)
RRAS2	22800	1.861161366
VTI1A	143187	1.861161366
REEP6	92840	1.86036693
DPEP1	1800	1.859969548
FAM98A	25940	1.859969548
SRRM1	10250	1.856702372
MDC1	9656	1.856612453
SEC31A	22872	1.856500776
ARAF	369	1.855591108
ASL	435	1.855192408
NFATC2IP	84901	1.855192408
FTL	2512	1.853886754
SIK3	23387	1.853197255
TBL2	26608	1.851998837
PTMS	5763	1.85155097
IRS1	3667	1.84949659
ITGBL1	9358	1.846794159
ARSE	415	1.845188935
EMG1	10436	1.844907741
C19orf53	28974	1.844278618
ZNF121	7675	1.843997497
RCC2	55920	1.843947452
RNF168	165918	1.843919861
UXS1	80146	1.843726145
BCCIP	56647	1.843699702
ALG12	79087	1.843696769
MCM5	4174	1.843648918
CCNH	902	1.843509065
LOC100190986	100190986	1.843462636
DYNLL2	140735	1.843356983
SPRY4	81848	1.843322313
NFATC2	4773	1.843309023
MIDN	90007	1.843215808
CWC15	51503	1.843201423
MAML1	9794	1.843185914
SMARCA5	8467	1.843140365
C16orf57	79650	1.843082627
NAA40	79829	1.842777745
MLLT10	8028	1.841570637
LRP11	84918	1.839959587
TM4SF1	4071	1.83874883
STIL	6491	1.838346737
CENPF	1063	1.837055483
DCTPP1	79077	1.833902077
ZFAND6	54469	1.833092483
DOCK5	80005	1.832687516
FTH1	2495	1.832658562
KIF3B	9371	1.831841925
SETD1A	9739	1.831709252

Gene	Entrez Gene ID	Log ratio RPKM(IGF2BP3)/RPKM(control IgG)
EPC2	26122	1.831471933
TMEM184B	25829	1.831316374
PIP5K1C	23396	1.829443681
GNPNAT1	64841	1.829037689
SNRNP27	11017	1.82822536
CAPRIN1	4076	1.827477887
MAPKAP1	79109	1.827311353
SLMAP	7871	1.826793306
IGFBP3	3486	1.826491523
NUFIP2	57532	1.825316205
BAZ2A	11176	1.825018899
PHACTR4	65979	1.823341762
CSNK1A1	1452	1.823135727
ADPRHL2	54936	1.822934048
KIAA0040	9674	1.821710215
SHROOM3	57619	1.818850561
WFDC2	10406	1.818441576
USP34	9736	1.817623258
ABCE1	6059	1.817168061
C6orf62	81688	1.817022425
KIF20A	10112	1.817016331
GTPBP1	9567	1.816923385
GHITM	27069	1.816742033
HES1	3280	1.816679501
ELK1	2002	1.816651692
MYEOV	26579	1.816304042
ASAH1	427	1.81393507
XPO7	23039	1.81393507
STAG3L1	54441	1.812703577
ICAM2	3384	1.812292845
FXC1	26515	1.810237431
LASS6	253782	1.808590988
QARS	5859	1.806089955
UNC50	25972	1.805705185
BCAR1	9564	1.805424034
SRGAP1	57522	1.805393072
CDC25A	993	1.805292456
CD24	100133941	1.805272883
NCRNA00188	125144	1.805051906
BCL9	607	1.804879608
SCNN1A	6337	1.804624078
SCNM1	79005	1.804053559
FAM102A	399665	1.803390587
TFCP2	7024	1.802813598
LSM14A	26065	1.80240004
CSTB	1476	1.802107692
PHF19	26147	1.801986364
TIMM50	92609	1.801986364
LOC202181	202181	1.801986364

Gene	Entrez Gene ID	Log ratio RPKM(IGF2BP3)/RPKM(control IgG)
PNKP	11284	1.801572569
SS18	6760	1.799365472
MKRN1	23608	1.798672679
CHCHD4	131474	1.797843067
MMADHC	27249	1.796597754
OSBPL10	114884	1.794935663
TMEM106B	54664	1.794519841
CCDC101	112869	1.793271654
NEURL	9148	1.793167002
SMARCC2	6601	1.792049293
DAZAP2	9802	1.790875876
EXOC6B	23233	1.790355014
ANKRD13B	124930	1.789103218
TAF13	6884	1.788268083
H19	283120	1.788268083
ZNF620	253639	1.787850334
KPNA6	23633	1.786819774
ST7	7982	1.784085143
IQSEC1	9922	1.78302472
MKI67	4288	1.782220516
ULK1	8408	1.780310099
TP53I13	90313	1.778629126
SLC25A46	91137	1.778208576
MPHOSPH9	10198	1.77707457
FAM168A	23201	1.776981273
RAB14	51552	1.776946192
PNPO	55163	1.776754912
MYST3	7994	1.776719327
FAM32A	26017	1.776710101
C12orf52	84934	1.776663921
DEDD2	162989	1.776660179
OPTN	10133	1.776610347
WSB2	55884	1.776607443
LPCAT1	79888	1.776605748
KIF20B	9585	1.776582573
YWHAH	7533	1.776548875
EIF4EBP1	1978	1.776543252
BRD7	29117	1.776541904
TNFRSF21	27242	1.776419034
EI24	9538	1.776370561
PDAP1	11333	1.776364715
PIGT	51604	1.776357438
TRAM1	23471	1.776304176
ARHGEF11	9826	1.776293987
RBM15	64783	1.77629028
GLYR1	84656	1.77628534
ANKRD52	283373	1.776283014
NFYC	4802	1.776267244
SLC35F2	54733	1.776251376

Gene	Entrez Gene ID	Log ratio RPKM(IGF2BP3)/RPKM(control IgG)
COX6C	1345	1.77617652
FARP1	10160	1.77615486
GINS4	84296	1.776133763
POLDIP2	26073	1.776108675
C12orf11	55726	1.77602712
PTPRE	5791	1.77602611
DFFA	1676	1.775901969
PSME1	5720	1.775875211
FXYD6	53826	1.775682702
SSU72	29101	1.775646062
RNF111	54778	1.773996325
LIPA	3988	1.773574423
EPS8	2059	1.773152397
BUB1	699	1.772307975
TSTD1	100131187	1.771040414
AKT2	208	1.769348599
ZNF562	54811	1.768925336
C12orf65	91574	1.763895506
CHD8	57680	1.761710784
EIF4G3	8672	1.760859637
IGF2BP2	10644	1.759938931
KIF2A	3796	1.756169328
PKIG	11142	1.756169328
C2orf3	6936	1.753176627
FAM91A1	157769	1.752748591
FRMD4A	55691	1.752320428
RNF10	9921	1.751892138
TNFRSF25	8718	1.751035177
PSAT1	29968	1.749681739
ZNF213	7760	1.74760223
ERRFI1	54206	1.746937453
STK4	6789	1.746797691
NCDN	23154	1.746668869
PRPF38B	55119	1.746373047
BCKDK	10295	1.746312766
ARCN1	372	1.745187716
PRKAR2A	5576	1.743299528
TSC22D4	81628	1.743299528
HCFC1	3054	1.743114543
TRAF3	7187	1.743071752
EXOSC4	54512	1.741574847
LARP1	23367	1.741566367
UBA1	7317	1.738781447
CCDC92	80212	1.737686761
PARN	5073	1.736821317
ZNF655	79027	1.735522177
PCSK7	9159	1.735187051
DYNLL1	8655	1.734920668
RAB43	339122	1.734884192

Gene	Entrez Gene ID	Log ratio RPKM(IGF2BP3)/RPKM(control IgG)
PRKACA	5566	1.734791857
BZW1	9689	1.734664961
TWISTNB	221830	1.734655433
CLCF1	23529	1.734408503
EPHB4	2050	1.734373815
PIK3R2	5296	1.73428525
LGALS8	3964	1.734197911
STMN1	3925	1.733987016
JMJD1C	221037	1.733354341
CCDC84	338657	1.730748629
SPRR3	6707	1.730313886
ARFGEF1	10565	1.729444007
LOC389791	389791	1.72900887
UBE2L3	7332	1.728174128
TSIX	9383	1.726831217
GALNT14	79623	1.725959235
PEG10	23089	1.725201231
HMGA2	8091	1.725198364
BCL9L	283149	1.725051378
CEP72	55722	1.724650272
ZNF839	55778	1.72377697
SKI	6497	1.723767025
SLC35E2	9906	1.723721928
SKIL	6498	1.722903139
ANKS1A	23294	1.720716243
TET2	54790	1.720278465
GREB1L	80000	1.719840555
RHOA	387	1.719428415
EHBP1	23301	1.718850007
PPARG	5468	1.718443511
PPP2R5D	5528	1.718324596
LDLR	3949	1.718062856
DDIT3	1649	1.717649008
ABCC9	10060	1.717205219
CALM3	808	1.716246784
PAX8	7849	1.71501475
LPP	4026	1.714135594
POMGNT1	55624	1.713255902
ADIPOR1	51094	1.711248786
EIF4H	7458	1.710117632
SSH1	54434	1.708849377
CPSF3L	54973	1.707966455
WEE1	7465	1.707524791
REST	5978	1.707082992
AGPAT1	10554	1.706404901
CNPY3	10695	1.706389776
BID	637	1.706382301
SPINT1	6692	1.706357608
BECN1	8678	1.706268797

Gene	Entrez Gene ID	Log ratio RPKM(IGF2BP3)/RPKM(control IgG)
HIST1H4D	8360	1.706213028
PGAM5	192111	1.706182103
SMARCC1	6599	1.706181068
EXOSC10	5394	1.706169078
RAVER1	125950	1.706168495
PFN2	5217	1.706132872
MRPS10	55173	1.706024351
SENP3	26168	1.706022841
ATF5	22809	1.706010609
TSKU	25987	1.705990158
CRAT	1384	1.70595289
NUP62	23636	1.705837363
SHC1	6464	1.705834693
CDK2AP1	8099	1.705814631
LAD1	3898	1.705810514
B4GALT2	8704	1.705645921
GNS	2799	1.705643983
SAE1	10055	1.704979407
NOL11	25926	1.699995744
C19orf55	148137	1.699995744
ACIN1	22985	1.698833571
POLDIP3	84271	1.697445134
SOCS7	30837	1.697329024
DCAF7	10238	1.697146486
NR1D1	9572	1.696884091
SLC7A5P1	81893	1.696439021
MRPL53	116540	1.696439021
ARFGEF2	10564	1.694842299
GNB1	2782	1.694764361
SORL1	6653	1.694199837
IL13RA1	3597	1.693765712
RNF114	55905	1.693319679
ITGB8	3696	1.691996148
GPRC5A	9052	1.691864539
CPSF6	11052	1.691542872
BCL2L1	598	1.691053671
TMEM33	55161	1.690640579
IQCE	23288	1.689746438
EPB41L1	2036	1.689117301
ZGPAT	84619	1.687597413
IL15RA	3601	1.687508661
FGD6	55785	1.686612577
NANS	54187	1.685715936
TMED7	51014	1.685302189
FURIN	5045	1.685193734
MICB	4277	1.684369929
LONP2	83752	1.683920981
CARS2	79587	1.683022665
RAPGEFL1	51195	1.681674142

Gene	Entrez Gene ID	Log ratio RPKM(IGF2BP3)/RPKM(control IgG)
GALNT10	55568	1.681495296
TARS	6897	1.680088507
SP2	6668	1.679874148
ROCK1	6093	1.679676385
KIAA1522	57648	1.679356808
ESAM	90952	1.678973308
PROCA1	147011	1.676267408
TMEM223	79064	1.673556424
KLHDC3	116138	1.672199019
VPS35	55737	1.670050835
CPSF7	79869	1.669833256
PHF3	23469	1.669816227
ZMAT2	153527	1.66974425
ZNF207	7756	1.669682177
EIF2A	83939	1.669674731
G6PC3	92579	1.669595947
RAB10	10890	1.669583214
ZNF664	144348	1.669567095
F2RL1	2150	1.66955965
PEX26	55670	1.66954865
GATAD2A	54815	1.669506902
ZIC2	7546	1.669498585
PICALM	8301	1.669474568
AIM1	202	1.669354688
SCAMP4	113178	1.669212387
ISOC2	79763	1.669188784
BAP1	8314	1.669162424
RASGRF2	5924	1.66766509
TUT1	64852	1.665847521
PLEKHG3	26030	1.664937877
RAB40C	57799	1.664937877
C21orf33	8209	1.663116867
VPS28	51160	1.663116867
KPNB1	3837	1.662233714
FERMT1	55612	1.660954535
NFRKB	4798	1.658554261
CTDSPL	10217	1.658097205
C20orf112	140688	1.65718266
PPP1CB	5500	1.657130642
NUCKS1	64710	1.656963598
SORT1	6272	1.656788933
TMC5	79838	1.65672517
KNTC1	9735	1.654435541
FANCI	55215	1.653977179
FAM83A	84985	1.65342262
CSK	1445	1.651223943
OSBP	5007	1.649385529
CBLC	23624	1.64800518
NETO2	81831	1.647786396

Gene	Entrez Gene ID	Log ratio RPKM(IGF2BP3)/RPKM(control IgG)
ALAS1	211	1.647385001
RSRC2	65117	1.647372536
LRFN4	78999	1.647347667
FSTL1	11167	1.647073096
NFE2L2	4780	1.647026172
NCOA5	57727	1.646905519
PSMC6	5706	1.646162657
WWTR1	25937	1.645401107
NEAT1	283131	1.642495228
CAMSAP1L1	23271	1.641083553
TAPBP	6892	1.640872625
SLC38A1	81539	1.640647109
PRCC	5546	1.639646173
TM9SF3	56889	1.639039173
ASNA1	439	1.63784206
DR1	1810	1.635986504
SLK	9748	1.635751335
CDV3	55573	1.634597871
DNAJC4	3338	1.634128558
CRCP	27297	1.634128558
INO80D	54891	1.633198686
ESCO1	114799	1.632733526
PTP4A1	7803	1.632190778
PPP2R2C	5522	1.632174699
IRF2BP2	359948	1.6321206
CD47	961	1.632066736
SMEK1	55671	1.631971761
PIM3	415116	1.631923981
CASC3	22794	1.63191778
FAM82A2	55177	1.631802755
NEK6	10783	1.631337144
ZDHHC9	51114	1.631337144
CEP170	9859	1.630555078
STK24	8428	1.630215279
DNTTIP1	116092	1.629939409
KHSRP	8570	1.629200396
GLE1	2733	1.629006833
AAK1	22848	1.629006833
PDHA1	5160	1.62878204
EIF2C2	27161	1.626819006
C1GALT1	56913	1.626672753
SH3D19	152503	1.626672753
ZYX	7791	1.626496001
ASH2L	9070	1.625738062
UPF2	26019	1.625738062
BMP4	652	1.624334889
PPFIBP1	8496	1.622461867
MEF2D	4209	1.621755958
B4GALT5	9334	1.621488377

Gene	Entrez Gene ID	Log ratio RPKM(IGF2BP3)/RPKM(control IgG)
MRPS15	64960	1.621293009
DNM1L	10059	1.621260517
CHMP1A	5119	1.621166948
DNM1	1759	1.620951415
TM9SF4	9777	1.620884275
DTX2	113878	1.619853147
RBMS2	5939	1.618286734
ARHGAP26	23092	1.617925829
CNOT6	57472	1.616357697
ITPK1	3705	1.615669837
ATRX	546	1.614003045
HOXA3	3200	1.613531653
LRP8	7804	1.612990833
DLST	1743	1.612880069
ZMYND8	23613	1.612808535
KDM6B	23135	1.612794188
SIN3A	25942	1.612369706
FAM73B	84895	1.612116552
RAB3IL1	5866	1.61117238
DHFR	1719	1.61022759
NSD1	64324	1.609492436
DAB2IP	153090	1.609044464
RYK	6259	1.608809243
SH3PXD2A	9644	1.6082666
SPC24	147841	1.6073895
MAPK1IP1L	93487	1.606870451
SMAD5	4090	1.606641271
TSFM	10102	1.606007419
EIF4EBP2	1979	1.605968359
PDCD2	5134	1.605494334
MPHOSPH8	54737	1.602171791
ATXN1L	342371	1.602171791
MLL4	9757	1.601668833
RPL38	6169	1.60163277
IGF2BP3	10643	1.601564468
ATP5C1	509	1.601276401
LARP4	113251	1.600953127
TMEM39A	55254	1.600745498
HMGB2	3148	1.598972676
NIN	51199	1.597293303
PPDPF	79144	1.59724083
AP2A2	161	1.595980979
FBXL18	80028	1.595503661
HIATL1	84641	1.595503661
LRRC59	55379	1.595432997
NOTCH2	4853	1.593931725
GAS2L3	283431	1.593544019
ATL2	64225	1.593114696
AKAP1	8165	1.591679417

Gene	Entrez Gene ID	Log ratio RPKM(IGF2BP3)/RPKM(control IgG)
CASP4	837	1.59072177
RNF139	11236	1.589763487
KCNK6	9424	1.589284107
STARD10	10809	1.589284107
AFF1	4299	1.587364991
RPL12	6136	1.585757155
NGRN	51335	1.585599734
POLR2D	5433	1.585443319
VTI1B	10490	1.585443319
NOL8	55035	1.584962501
MSH6	2956	1.584481522
BRF1	2972	1.583037624
RUSC2	9853	1.583037624
PPRC1	23082	1.582290842
ATF7IP	55729	1.582074221
CBWD1	55871	1.581110175
AP1S3	130340	1.579662897
TRIP12	9320	1.579197478
SP140L	93349	1.579180148
TYW3	127253	1.579180148
UQCR11	10975	1.578214165
DCAF12	25853	1.578214165
RASSF3	283349	1.578214165
UBE2J1	51465	1.577247536
CHFR	55743	1.577247536
CHD1L	9557	1.575796375
C1orf144	26099	1.574496142
MED25	81857	1.573859222
ANLN	54443	1.572260647
SGMS2	166929	1.567633684
ELAVL1	1994	1.566084491
TCTEX1D2	255758	1.566084491
RNF214	257160	1.566084491
EIF1B	10289	1.565597176
SERPINB8	5271	1.56315813
CASP2	835	1.562229164
REV1	51455	1.560225822
NDEL1	81565	1.560225822
SNAP47	116841	1.55924706
ZNF689	115509	1.558267634
C6orf141	135398	1.556306784
CAMTA2	23125	1.555816155
HIST1H2AJ	8331	1.555085459
MAP2K3	5606	1.554740168
SPDYE7P	441251	1.554695061
RBM4	5936	1.554644627
SHARPIN	81858	1.554643567
STAP2	55620	1.554588852
TERF2	7014	1.554559756

Gene	Entrez Gene ID	Log ratio RPKM(IGF2BP3)/RPKM(control IgG)
CST1	1469	1.55452018
SLC38A7	55238	1.55450521
GTF2A1	2957	1.554486461
UCHL5	51377	1.554486187
POP1	10940	1.554471419
OAZ1	4946	1.554445004
COPS7A	50813	1.554440556
TGS1	96764	1.554421917
SAMD10	140700	1.554413437
FAM199X	139231	1.554407923
LSM14B	149986	1.554406283
C11orf2	738	1.554399594
TAF1D	79101	1.554386425
CFB	629	1.554367091
SFXN3	81855	1.554355937
THUMPD1	55623	1.554343266
NFKBIA	4792	1.554305479
DBNL	28988	1.554302771
RSRC1	51319	1.554294614
C20orf20	55257	1.55427677
JAK1	3716	1.55425637
SCAND1	51282	1.554249956
FAM96B	51647	1.554234066
TNFRSF12A	51330	1.554226463
CTNNAL1	8727	1.554220162
BTBD2	55643	1.554209843
MOBKL1B	55233	1.55420836
POLR2J	5439	1.554206209
MRPL35	51318	1.554204823
THOC7	80145	1.554194096
ETNK1	55500	1.554178058
ARL2	402	1.554176375
PPP1CC	5501	1.554139577
ATPIF1	93974	1.55412969
ACBD6	84320	1.554118033
PSMB10	5699	1.554116534
GPX2	2877	1.554109153
PPP2R5C	5527	1.554101537
PKP4	8502	1.554091289
CLIP1	6249	1.554083844
MAX	4149	1.554082627
CBX1	10951	1.554073055
WHSC1L1	54904	1.554070943
SIPA1L3	23094	1.554068493
F11R	50848	1.554064542
NAA50	80218	1.554060449
FAM49B	51571	1.554059519
RPS28	6234	1.554057873
NADSYN1	55191	1.554051944

Gene	Entrez Gene ID	Log ratio RPKM(IGF2BP3)/RPKM(control IgG)
GLA	2717	1.55404723
SLC9A3R2	9351	1.554040035
ADAM10	102	1.554026334
SFXN1	94081	1.554017517
MYL12A	10627	1.554017228
BRIX1	55299	1.554013912
FADD	8772	1.553996495
LIF	3976	1.553984791
BRD9	65980	1.553980889
MBD2	8932	1.553965455
DHCR24	1718	1.55396201
USP7	7874	1.553959419
SETD8	387893	1.553956775
PHLDA2	7262	1.553923009
HIST1H2AL	8332	1.553909919
RHOB	388	1.553899164
STT3B	201595	1.553897427
CHEK1	1111	1.553883385
PSMD12	5718	1.553882129
FAH	2184	1.553876176
SELS	55829	1.553874823
MPZL1	9019	1.553871309
SLC35E1	79939	1.553851968
DDAH2	23564	1.553846833
YY1	7528	1.553820279
SRFBP1	153443	1.553819118
GOLIM4	27333	1.553797179
TRMT11	60487	1.553795541
ATF4	468	1.553795183
FAM162A	26355	1.553793801
DNAJA3	9093	1.553791951
PSMD11	5717	1.553778755
AIDA	64853	1.553773693
HIST1H4L	8368	1.553767879
NOL9	79707	1.553728915
JUND	3727	1.55371479
FAM193A	8603	1.553708848
XAB2	56949	1.553708517
SH3BP4	23677	1.55370832
NUDT5	11164	1.553663879
RPUSD1	113000	1.553653028
SCARNA5	677775	1.553632964
SEPT6	23157	1.553585041
C11orf1	64776	1.553583056
S100A11	6282	1.553507148
GNG12	55970	1.553484269
DNAJC3	5611	1.553439059
UBE2Q1	55585	1.553422723
GGA3	23163	1.553415434

Gene	Entrez Gene ID	Log ratio RPKM(IGF2BP3)/RPKM(control IgG)
BRP44L	51660	1.553404432
DIMT1L	27292	1.553392422
ADI1	55256	1.553376673
TRMT112	51504	1.553359806
DDX54	79039	1.553291889
OTUD4	54726	1.553204484
CREG1	8804	1.553094447
YTHDF2	51441	1.552377071
FAM131C	348487	1.551392968
ZNF777	27153	1.548929769
CALB2	794	1.547943311
NACA	4666	1.547390735
ENAH	55740	1.546971718
HUWE1	10075	1.546713329
C16orf45	89927	1.544979883
SOCS3	9021	1.54399072
FUT8	2530	1.543000877
HDLBP	3069	1.542783936
PANK2	80025	1.542010356
MED10	84246	1.541514839
CHMP2A	27243	1.541205192
TMEM63A	9725	1.541019153
GTF2H4	2968	1.538538164
SH3BGRL	6451	1.538538164
RALGPS2	55103	1.538538164
IP6K1	9807	1.538041453
ATP6V0A1	535	1.537047519
PPP5C	5536	1.537047519
RUSC1	23623	1.537047519
C19orf54	284325	1.534061602
MYADM	91663	1.533873729
RNPEP	6051	1.532067552
LRRC37A4	55073	1.531069493
LRRC57	255252	1.530570204
PXN	5829	1.529713942
GALNT9	50614	1.529571108
ZNF362	149076	1.529571108
PIK3C2A	5286	1.5290713
TRIM33	51592	1.5290713
TGFA	7039	1.528950475
PPARD	5467	1.527878969
SGSH	6448	1.527070336
TCF20	6942	1.526896173
MGAT5	4249	1.52656966
TOP2B	7155	1.52656966
NFKBIL1	4795	1.526068812
C18orf21	83608	1.526068812
FAM57A	79850	1.524063676
TPRG1L	127262	1.524063676

Gene	Entrez Gene ID	Log ratio RPKM(IGF2BP3)/RPKM(control IgG)
MSL1	339287	1.522557993
KIAA0196	9897	1.522055749
YWHAZ	7534	1.521108045
ANKRD40	91369	1.520547968
LSM3	27258	1.520045024
ATMIN	23300	1.519541905
FBLIM1	54751	1.519541905
CCDC88C	440193	1.519338916
SLC7A6	9057	1.518535139
KLK10	5655	1.518449227
SYNE2	23224	1.518074645
CDK10	8558	1.517023672
ST14	6768	1.516729131
DNPEP	23549	1.516519498
CLDN2	9075	1.516164322
PPWD1	23398	1.514501036
NCOA3	8202	1.513747795
XPO6	23214	1.513241081
LOC100289019	100289019	1.512479747
LGR4	55366	1.510961919
ERAP2	64167	1.510961919
NDST1	3340	1.509600501
FAM107B	83641	1.50956699
WAPAL	23063	1.509442493
TRIB3	57761	1.509442493
FBXO28	23219	1.508428653
C1orf159	54991	1.506906555
CEP57	9702	1.50589093
RPTOR	57521	1.505411936
MFSD10	10227	1.505382849
TOMM40	10452	1.505169762
SGK223	157285	1.505004385
FBXO7	25793	1.503857533
LLGL2	3993	1.503348735
AURKA	6790	1.502078951
EXPH5	23086	1.501821265
RACGAP1	29127	1.501163274
JAG1	182	1.500292177
CCDC41	51134	1.500292177
ANKLE2	23141	1.499896309
IL34	146433	1.49978212
SURF4	6836	1.499610904
MRPS18A	55168	1.495695163
NLK	51701	1.494671612
CDC7	8317	1.494159564
PARG	8505	1.492109553
SNX27	81609	1.492109553
TMEM201	199953	1.491607092
ACSL3	2181	1.491596594

Gene	Entrez Gene ID	Log ratio RPKM(IGF2BP3)/RPKM(control IgG)
C1orf116	79098	1.491596594
FYCO1	79443	1.491596594
ARMC7	79637	1.491596594
SPTLC1	10558	1.491074734
CNOT8	9337	1.49057013
SCAP	22937	1.49057013
BCORL1	63035	1.490056624
NDUFV3	4731	1.489542936
PARD6B	84612	1.489542936
YAP1	10413	1.488141212
ACAT1	38	1.487486349
GNA15	2769	1.487486349
FAM192A	80011	1.486971744
RNF169	254225	1.486971744
ERBB2	2064	1.486456956
PEX19	5824	1.485426827
TAF4	6874	1.485426827
SMCR7L	54471	1.484395963
COBRA1	25920	1.483867999
ZNF638	27332	1.483734957
CCDC99	54908	1.483653006
PPT1	5538	1.483651514
GRB2	2885	1.483632344
LMO7	4008	1.482848283
ANKRD13D	338692	1.482848283
TMEM87A	25963	1.482559077
DGKA	1606	1.48244383
ITGA6	3655	1.482341931
EPN3	55040	1.481298942
SH3YL1	26751	1.480265122
ENDOD1	23052	1.478195258
CALM1	801	1.47815431
BRD8	10902	1.476640909
TAF2	6873	1.475084883
LRRC61	65999	1.474565835
TMEM8B	51754	1.474046599
AXL	558	1.473640951
ZNF623	9831	1.473527177
FAM171A1	221061	1.473527177
TIMM9	26520	1.473007568
UBQLN1	29979	1.472198126
TPP2	7174	1.472016707
PRPS1	5631	1.471967788
PLEKHG6	55200	1.471967788
RAB2A	5862	1.471923413
CHRAC1	54108	1.471780011
GTF3C2	2976	1.471677772
DDA1	79016	1.471542365
SRP68	6730	1.471447616

Gene	Entrez Gene ID	Log ratio RPKM(IGF2BP3)/RPKM(control IgG)
RIN3	79890	1.47135037
FAM83B	222584	1.471268975
NT5C2	22978	1.471244605
WHSC1	7468	1.471001857
UBE2D3	7323	1.47078588
TMEM194A	23306	1.469885976
RFX7	64864	1.469885976
ERICH1	157697	1.469885976
GGPS1	9453	1.469521257
MORC4	79710	1.469037092
GNAQ	2776	1.468843943
ZZZ3	26009	1.468322644
TENC1	23371	1.466757616
PTPN11	5781	1.466602251
CCDC120	90060	1.466485264
NR2F6	2063	1.466235562
C1orf123	54987	1.466235562
KCTD10	83892	1.465190888
DFNB31	25861	1.464668267
DHX37	57647	1.464005697
GOLGA3	2802	1.463679007
CLCN3	1182	1.463622457
ARHGEF7	8874	1.463622457
KRT80	144501	1.463356831
CEACAM6	4680	1.463325382
TMEM48	55706	1.461528559
SLC30A9	10463	1.45995614
NFU1	27247	1.459431619
TERF1	7013	1.458906907
C1orf130	400746	1.458906907
LRCH1	23143	1.45785691
HAUS8	93323	1.45785691
SLC25A44	9673	1.456280482
AP1AR	55435	1.455754622
SENP1	29843	1.455228571
C9orf78	51759	1.455228571
CCNL1	57018	1.454897285
ZNF146	7705	1.454895293
RBM17	84991	1.454872249
CLN6	54982	1.454746131
BOK	666	1.454735123
CYP51A1	1595	1.454713273
KLHL18	23276	1.454702328
EDC4	23644	1.454702328
PEAR1	375033	1.454659053
PLCB3	5331	1.454650996
AXIN1	8312	1.454604241
IDH3A	3419	1.454412826
MCM6	4175	1.454408168

Gene	Entrez Gene ID	Log ratio RPKM(IGF2BP3)/RPKM(control IgG)
SLMO2	51012	1.454340176
NAA25	80018	1.454175893
DENR	8562	1.454061976
SETD2	29072	1.453774837
HELZ	9931	1.453122447
EPC1	80314	1.452595435
C3	718	1.451540833
ZNF480	147657	1.451013243
AP2B1	163	1.450855953
SREBF2	6721	1.44999804
BAK1	578	1.447843644
ADPGK	83440	1.447843644
ACER3	55331	1.446785562
THSD4	79875	1.445726703
PLA2G16	11145	1.445533058
TPR	7175	1.445504565
GPBP1	65056	1.444960236
APOL1	8542	1.444338056
SOLH	6650	1.44359567
TMBIM1	64114	1.443508753
PHLDB1	23187	1.44333667
GPATCH4	54865	1.442888766
RAP1GAP	5909	1.442545456
NUPL1	9818	1.442334378
RASSF7	8045	1.442032896
C1QTNF6	114904	1.440952198
TNIP1	10318	1.440218246
EIF4G2	1982	1.439843573
MXRA7	439921	1.439357178
ADRBK1	156	1.439050396
SH3BP1	23616	1.437760394
ETS1	2113	1.437519599
KIF5B	3799	1.437319746
HEXIM1	10614	1.437227739
FMNL3	91010	1.437227739
CREB3L2	64764	1.436315208
ARFIP2	23647	1.436161839
SNHG12	85028	1.435095152
GNAI1	2770	1.434561512
HMMR	3161	1.432018262
RNF130	55819	1.431890348
GOSR1	9527	1.430083056
CUL4B	8450	1.42921423
CNTROB	116840	1.42891831
TNPO1	3842	1.428691906
LEMD2	221496	1.428652343
SELP	6403	1.428586654
NRAS	4893	1.428504651
SEC14L1	6397	1.428473013

Gene	Entrez Gene ID	Log ratio RPKM(IGF2BP3)/RPKM(control IgG)
EARS2	124454	1.428334322
MEGF6	1953	1.428222611
PITRM1	10531	1.428213584
KHDRBS1	10657	1.428209286
TFPI	7035	1.428187658
YY1AP1	55249	1.428074858
GOLGA5	9950	1.427906845
PIP4K2A	5305	1.42787355
PARD3	56288	1.427772256
SC4MOL	6307	1.427606173
C15orf42	90381	1.427606173
KIAA2018	205717	1.427606173
C3orf72	401089	1.427606173
IQCB1	9657	1.427069755
LOC731275	731275	1.426706979
NFE2L3	9603	1.425996321
DHX38	9785	1.424922088
DHTKD1	55526	1.424922088
ANAPC1	64682	1.424922088
YBX1	4904	1.424205233
COL27A1	85301	1.421155961
TCEA1	6917	1.420274148
MAP4	4134	1.420241839
MNT	4335	1.420078116
FAM92A1	137392	1.420078116
ALCAM	214	1.419564
SERGEF	26297	1.419538892
ERC1	23085	1.419530685
LMTK2	22853	1.418999465
ANKRD28	23243	1.418459838
MRPS35	60488	1.418459838
HSF1	3297	1.416837666
HDGF	3068	1.416698231
HP1BP3	50809	1.41655214
CAST	831	1.416546534
ZNF629	23361	1.416311056
BIRC6	57448	1.416048144
HIVEP2	3097	1.414135533
TET3	200424	1.41384845
TARBP2	6895	1.413594082
KIAA0754	643314	1.413458194
TOP3A	7156	1.413052429
MKL1	57591	1.413052429
C10orf28	27291	1.41196851
HAUS1	115106	1.411426246
SP3	6670	1.410163525
RBM26	64062	1.40990726
GPR35	2859	1.409798228
C2orf18	54978	1.409731006

Gene	Entrez Gene ID	Log ratio RPKM(IGF2BP3)/RPKM(control IgG)
CDKN1A	1026	1.40948301
ZNF687	57592	1.407624676
HAVCR1	26762	1.407416272
IFRD1	3475	1.40653667
LMBR1	64327	1.40599236
C17orf37	84299	1.40599236
DCAF15	90379	1.40599236
CCDC85B	11007	1.405256478
ETV4	2118	1.404903122
HNRNPC	3183	1.40332282
THAP11	57215	1.402176425
C10orf18	54906	1.401871729
EIF2C1	26523	1.401869399
GYS1	2997	1.401590368
ADH5	128	1.401084302
NEDD4L	23327	1.401084302
ARMC1	55156	1.401084302
PRMT6	55170	1.401084302
DRG2	1819	1.399991351
SEPHS2	22928	1.399991351
CHKA	1119	1.399751934
ASXL1	171023	1.398152396
LETM1	3954	1.397974614
OTUB2	78990	1.397952683
LRRC47	57470	1.397255346
UBTF	7343	1.397243241
FAM168B	130074	1.395277546
SEMA3C	10512	1.395259631
ATP9A	10079	1.394514142
DENND4B	9909	1.393965276
SLC48A1	55652	1.393965276
ASH1L	55870	1.393696613
MTDH	92140	1.393651047
ZNF706	51123	1.392866916
TPT1	7178	1.392344061
CHD1	1105	1.39176772
CEP70	80321	1.390667686
FAM59A	64762	1.390117354
SCRN1	9805	1.389503437
PRDM4	11108	1.38901606
DST	667	1.388548655
KANK2	25959	1.388465097
UACA	55075	1.387913924
TMEM189	387521	1.386810946
SYT12	91683	1.386259141
SPAG9	9043	1.384705218
TRMT2A	27037	1.38466385
IL18	3606	1.384602458
NIPSNAP1	8508	1.384540072

Gene	Entrez Gene ID	Log ratio RPKM(IGF2BP3)/RPKM(control IgG)
PKMYT1	9088	1.384537003
TNFSF10	8743	1.384504797
TXNDC11	51061	1.384480032
BCYRN1	618	1.38447175
SERINC3	10955	1.384415211
CIRH1A	84916	1.384312806
MLXIP	22877	1.384293976
PDZD11	51248	1.384250508
TRIM27	5987	1.384216323
USP36	57602	1.384203671
WDR43	23160	1.384182868
LTV1	84946	1.384162944
YME1L1	10730	1.384102117
SDHD	6392	1.38410165
C12orf75	387882	1.384080687
BSDC1	55108	1.384043603
GTF2F2	2963	1.384036677
ZNF358	140467	1.38403567
DNAJB12	54788	1.384020866
POLD4	57804	1.383994737
GPS2	2874	1.383966053
SCAF1	58506	1.383953048
RASSF10	644943	1.383939452
PRSS3	5646	1.383925669
RABGGTB	5876	1.383881967
HPRT1	3251	1.383840143
KCNN4	3783	1.383786565
UFM1	51569	1.38369694
NIPA2	81614	1.38357687
IFRD2	7866	1.383477876
OCLN	100506658	1.383390931
ARHGAP11A	9824	1.383348078
MLLT3	4300	1.38294387
RELT	84957	1.382390583
LDB1	8861	1.381283373
MTMR2	8898	1.380175312
RBM7	10179	1.380175312
TMEM41B	440026	1.379620962
KIAA1731	85459	1.379066399
NAV2	89797	1.377893743
PIGL	9487	1.376846014
PWWP2B	170394	1.376846014
FAM63B	54629	1.376290383
MYO1C	4641	1.376138953
PAPSS2	9060	1.37517848
FGFR1	2260	1.374646161
SLC22A18	5002	1.374065718
LIMK1	3984	1.371646835
UHRF1BP1	54887	1.371280054

Gene	Entrez Gene ID	Log ratio RPKM(IGF2BP3)/RPKM(control IgG)
NUP85	79902	1.371280054
PPP6C	5537	1.370722275
ARID3B	10620	1.370722275
LOC100131726	100131726	1.368489001
OAS3	4940	1.367966523
KIAA0368	23392	1.367850814
HECTD1	25831	1.367790621
TNRC6A	27327	1.367623307
UBXN4	23190	1.367415143
BIVM	54841	1.367371066
RBM12	10137	1.367371066
TBRG1	84897	1.366252264
KIAA1549	57670	1.365692537
BBX	56987	1.365132593
BAG1	573	1.364572432
TFAM	7019	1.364572432
CENPN	55839	1.364572432
CXCL16	58191	1.364572432
TTPAL	79183	1.364572432
MYBL2	4605	1.364309695
YTHDC1	91746	1.364012054
PLXND1	23129	1.36232961
TAF15	8148	1.361970168
HLA-DRB1	3123	1.361768359
CENPV	201161	1.361768359
SMC5	23137	1.361747797
RNF216	54476	1.361687225
SERINC5	256987	1.361422351
GIGYF1	64599	1.361074671
SLC25A39	51629	1.361008383
LACTB2	51110	1.360645202
TSC22D2	9819	1.35952117
MYO19	80179	1.357850643
KIAA0913	23053	1.357270476
DIS3L2	129563	1.357270476
SLC37A2	219855	1.356341164
NPLOC4	55666	1.356270613
VPS18	57617	1.355580147
SPSB1	80176	1.355580147
TMEM14C	51522	1.354452161
TMEM164	84187	1.353408219
RDBP	7936	1.353323291
C17orf88	23591	1.353323291
TRRAP	8295	1.352624892
SFPQ	6421	1.352443641
PARP10	84875	1.350497247
ARL8A	127829	1.350497247
ZBTB4	57659	1.349931373
SCRN2	90507	1.34879896

Gene	Entrez Gene ID	Log ratio RPKM(IGF2BP3)/RPKM(control IgG)
CHUK	1147	1.348232419
RAI14	26064	1.348072068
TPM4	7171	1.347790158
NVL	4931	1.347717425
CDR2L	30850	1.347708898
PDE8A	5151	1.347665656
GAS5	60674	1.347629466
LUC7L3	51747	1.34761809
EPN1	29924	1.347504342
TRIM11	81559	1.347460513
HDAC7	51564	1.347416136
KDELC2	143888	1.347356026
GJB3	2707	1.347352871
BRD2	6046	1.347243909
CPT1A	1374	1.347230097
ZNF282	8427	1.347208026
MARVELD1	83742	1.347202496
CSPP1	79848	1.347172532
DGCR8	54487	1.347114492
SMARCAD1	56916	1.347098671
CD109	135228	1.347098671
KLHL29	114818	1.347048865
STK40	83931	1.346989016
MAFG	4097	1.346802764
SYNJ2BP	55333	1.345396375
RHPN2	85415	1.345396375
NEU3	10825	1.344828497
C10orf118	55088	1.344828497
MEN1	4221	1.341416524
GOLM1	51280	1.340524051
PRPF4B	8899	1.339707507
NIPBL	25836	1.339707507
HEBP1	50865	1.336283388
CCAR1	55749	1.335668152
HIBADH	11112	1.333996118
CHD9	80205	1.333996118
COTL1	23406	1.333933083
CCDC142	84865	1.333423734
OPA1	4976	1.332851122
CNOT3	4849	1.331835646
ITSN1	6453	1.331587075
CAP1	10487	1.331547624
CD55	1604	1.331510398
TJP2	9414	1.331322498
TBC1D13	54662	1.331316599
COL6A1	1291	1.33130458
PSME3	10197	1.331205908
KIF4A	24137	1.331007389
ZHX2	22882	1.3305584

Gene	Entrez Gene ID	Log ratio RPKM(IGF2BP3)/RPKM(control IgG)
PAAF1	80227	1.3305584
TTC37	9652	1.32998465
TTLL5	23093	1.32998465
C12orf49	79794	1.32998465
KLHL5	51088	1.329410671
PDDC1	347862	1.329410671
LOC541471	541471	1.329410671
PRELID1	27166	1.328836464
C12orf56	115749	1.328836464
DLG1	1739	1.328388144
DOCK1	1793	1.327687364
NKIRAS2	28511	1.326537348
RGS19	10287	1.325961996
YIPF6	286451	1.325386415
MED26	9441	1.324234562
SRI	6717	1.322505058
MAST2	23139	1.32247002
SYNJ2	8871	1.322352355
STK35	140901	1.32233159
CUEDC2	79004	1.321928095
EED	8726	1.321350901
FAT1	2195	1.320716625
MUC1	4582	1.320195821
ANAPC16	119504	1.320195821
WASF2	10163	1.319505433
LIME1	54923	1.319039816
TRPS1	7227	1.318461465
SULT2B1	6820	1.317882883
CLPTM1	1209	1.317470415
SF3A1	10291	1.317172824
RAPGEF1	2889	1.316746728
DPY30	84661	1.316725022
STK11IP	114790	1.316725022
SHMT1	6470	1.315128263
GCFC1	94104	1.313826296
LY6D	8581	1.313245852
KIAA0319L	79932	1.313245852
C7orf40	285958	1.313245852
FLJ45340	402483	1.312721044
SNX18	112574	1.312084262
SMAD3	4088	1.311988624
ENTPD6	955	1.311201688
TFPT	29844	1.310921735
ADAR	103	1.310489116
MFF	56947	1.310340121
USP48	84196	1.310340121
PLEKHM2	23207	1.309969723
LOC283070	283070	1.309758271
CMPK1	51727	1.309523982

Gene	Entrez Gene ID	Log ratio RPKM(IGF2BP3)/RPKM(control IgG)
RG9MTD1	54931	1.308011315
SETD1B	23067	1.307925235
NPEPPS	9520	1.307684164
VCL	7414	1.306674605
MT1X	4501	1.30626224
PSD3	23362	1.30626224
RIF1	55183	1.305836523
ANAPC11	51529	1.304511042
STK3	6788	1.303926836
MAP4K4	9448	1.30344788
GAB2	9846	1.302757716
POLG	5428	1.301587647
NT5E	4907	1.300971376
GPN1	11321	1.300416628
NSFL1C	55968	1.299830762
TM9SF1	10548	1.298658316
C4orf27	54969	1.298658316
OSMR	9180	1.298442854
RBL1	5933	1.297484916
N4BP2L2	10443	1.297484916
PARP14	54625	1.296897858
DHX33	56919	1.296310561
HPGD	3248	1.296094022
LRRC8D	55144	1.295952389
UBIAD1	29914	1.295723025
KIFC2	90990	1.295723025
NAB1	4664	1.294547234
ATF1	466	1.293958979
RNF213	57674	1.293370484
FAM127C	441518	1.293370484
C16orf59	80178	1.292781749
PKN2	5586	1.292192774
PPP2R4	5524	1.291836616
ACAT2	39	1.29175066
CDC42EP3	10602	1.291514425
STOML2	30968	1.291473817
RNASEH2C	84153	1.291432749
TRIP6	7205	1.291417546
PLSCR3	57048	1.291417546
C3orf75	54859	1.291416207
ERGIC2	51290	1.291393727
KIF23	9493	1.291348687
GAPVD1	26130	1.291336288
DGCR6L	85359	1.29129296
COPS5	10987	1.29127659
PHC2	1912	1.291241073
SURF2	6835	1.291218435
PCDH1	5097	1.29121684
PAK6	56924	1.29121684

Gene	Entrez Gene ID	Log ratio RPKM(IGF2BP3)/RPKM(control IgG)
TMED4	222068	1.291215738
SLC35C2	51006	1.291172567
HS3ST1	9957	1.29117144
ADORA2B	136	1.291145113
SPG21	51324	1.291120399
ARHGEF4	50649	1.291119556
EP400	57634	1.291116277
ATP5I	521	1.29107813
CTSB	1508	1.291068096
HNRNPA0	10949	1.291065617
AP1M1	8907	1.291060817
EXOSC8	11340	1.291048782
HIPK1	204851	1.291048782
GINS2	51659	1.291024684
ITFG2	55846	1.290988467
C7orf59	389541	1.290984153
AP2A1	160	1.290969895
GTSE1	51512	1.290958701
SYMPK	8189	1.290944191
C16orf42	115939	1.290938227
PRKAG1	5571	1.290922038
GGNBP2	79893	1.290914027
CETN2	1069	1.290894558
GPBP1L1	60313	1.29088969
YDJC	150223	1.290884714
C9orf16	79095	1.290883629
RSC1A1	6248	1.290878369
SRP19	6728	1.290873939
KIF22	3835	1.290856002
MLKL	197259	1.290824012
LRPAP1	4043	1.290820593
SLC16A5	9121	1.290806426
HTRA1	5654	1.290803238
TSPAN31	6302	1.290794063
PPP1R10	5514	1.290779396
MRPL23	6150	1.290768995
ATP6V1F	9296	1.290752199
NCLN	56926	1.290747921
COPS6	10980	1.29074339
PSMC1	5700	1.290730407
AGPAT9	84803	1.290729551
ZC3H11A	9877	1.290685394
FAF1	11124	1.290670483
DYNC1LI2	1783	1.290669687
MRPL30	51263	1.290663881
CXCL2	2920	1.290658654
AHRR	57491	1.290599154
SAFB2	9667	1.290574252
NUDT21	11051	1.290534771

Gene	Entrez Gene ID	Log ratio RPKM(IGF2BP3)/RPKM(control IgG)
YIPF2	78992	1.290521834
MRPL10	124995	1.290460762
PUM2	23369	1.290424404
FAM129A	116496	1.290424404
FAM120B	84498	1.290392521
ESF1	51575	1.29038623
HACL1	26061	1.290289414
CLK3	1198	1.290219235
LPHN2	23266	1.289834465
C10orf58	84293	1.289244285
SATB2	23314	1.2880632
RANBP10	57610	1.2880632
ADRB2	154	1.287472295
TNFRSF1B	7133	1.287472295
MST4	51765	1.286881148
RBM27	54439	1.286289758
MXI1	4601	1.285698126
TCFL5	10732	1.283921772
RNF182	221687	1.283921772
PLEKHM1	9842	1.283329168
EFTUD1	79631	1.283329168
HDAC5	10014	1.28273632
MARK4	57787	1.282143229
BPNT1	10380	1.281549893
QRFP	347148	1.280956314
LOC728190	728190	1.280956314
BDP1	55814	1.28036249
DPY19L2P2	349152	1.27857955
MARK3	4140	1.277453466
LITAF	9516	1.277389699
C9orf156	51531	1.276198865
MLEC	9761	1.275644736
DGAT1	8694	1.275603079
SPIN1	10927	1.274410769
FSCN1	6624	1.274174963
TBC1D9B	23061	1.273752922
UBASH3B	84959	1.273732405
CAV1	857	1.272838143
SHANK2	22941	1.272647192
KDM2A	22992	1.272592806
TTC35	9694	1.272023189
TAOK1	57551	1.271559937
CEP350	9857	1.271483596
FICD	11153	1.271425676
DHODH	1723	1.270827916
SNCG	6623	1.270827916
LOC646762	646762	1.270229907
PTBP1	5725	1.269842557
GTF2H1	2965	1.269033146

Gene	Entrez Gene ID	Log ratio RPKM(IGF2BP3)/RPKM(control IgG)
NUP188	23511	1.268863276
KIAA0649	9858	1.268803315
LARGE	9215	1.268434394
MPHOSPH10	10199	1.267835392
MON1B	22879	1.267835392
KIAA0556	23247	1.267835392
ZNF830	91603	1.267835392
GCAT	23464	1.267236142
NF1	4763	1.266636643
MACF1	23499	1.266289218
NCOR2	9612	1.266181755
SNTB1	6641	1.265436896
MARK2	2011	1.265023742
SCYL1	57410	1.26485312
SUV420H1	51111	1.264836648
NID1	4811	1.264672615
GPR108	56927	1.264236151
TNPO2	30000	1.263727843
C1orf174	339448	1.263635404
RNASET2	8635	1.262433158
CHML	1122	1.261831659
POU5F1	5460	1.261831659
PTGR1	22949	1.261229909
BDH1	622	1.260025656
ARRDC3	57561	1.260025656
MDM2	4193	1.258872736
PCDH7	5099	1.25882162
KIF18B	146909	1.258721727
AMOTL1	154810	1.258570371
PKP3	11187	1.258569282
CTBP1	1487	1.258374
SF3B4	10262	1.258351979
NFIL3	4783	1.25821739
C7orf50	84310	1.258212897
MYST4	23522	1.258091356
DECR1	1666	1.257010618
C3orf31	132001	1.255802837
SON	6651	1.255785267
FAM48A	55578	1.255198566
KCTD14	65987	1.255198566
KIF3C	3797	1.253989266
KIAA1217	56243	1.253384236
CCDC103	388389	1.253384236
PCM1	5108	1.252778952
RNF115	27246	1.252173413
CLIP2	7461	1.251951128
MAFK	7975	1.250961574
RPS6KA1	6195	1.250767066
ARF3	377	1.249961973

Gene	Entrez Gene ID	Log ratio RPKM(IGF2BP3)/RPKM(control IgG)
C6orf155	79940	1.249748715
PITPNA	5306	1.249400403
SLC35A4	113829	1.249342392
ZCCHC14	23174	1.249326151
DGKZ	8525	1.249261556
STC2	8614	1.249179211
ZKSCAN1	7586	1.24915641
BNIP2	663	1.249141903
HNRNPL	3191	1.249108762
FLOT2	2319	1.249076256
C22orf13	83606	1.248849462
INPP5A	3632	1.247927513
NRIP1	8204	1.247319935
UBE2L6	9246	1.246712101
SNX9	51429	1.246712101
NMNAT1	64802	1.246712101
MAGEF1	64110	1.245495663
FBXO31	79791	1.245495663
CDK5RAP1	51654	1.245274496
NDUFA8	4702	1.244887059
TRAPPC3	27095	1.244887059
ERCC6L	54821	1.244278199
DDX17	10521	1.244076363
SULF2	55959	1.243851937
SORD	6652	1.243669081
ABR	29	1.243259223
SCHIP1	29970	1.242450074
TAF6	6878	1.241433144
RPS6KA4	8986	1.240668236
DUSP4	1846	1.240068906
SLTM	79811	1.239867649
SYNCRIP	10492	1.239661175
C20orf177	63939	1.239398042
FBXL14	144699	1.239398042
LRSAM1	90678	1.238832479
UGP2	7360	1.23878686
SCYL2	55681	1.238175419
SDHC	6391	1.238159737
DMTF1	9988	1.237955626
MBOAT2	129642	1.237563718
C3orf39	84892	1.236339539
IGSF3	3321	1.23572706
C14orf102	55051	1.23572706
CCDC109B	55013	1.23511432
AIF1L	83543	1.234501321
UBE2I	7329	1.233166893
APOL2	23780	1.232660757
RC3H2	54542	1.232540377
EID1	23741	1.232433737

Gene	Entrez Gene ID	Log ratio RPKM(IGF2BP3)/RPKM(control IgG)
HIST1H2AG	8969	1.232420927
C11orf9	745	1.232244955
TBC1D2	55357	1.232160741
HNRNPA1	3178	1.232146429
APEX1	328	1.232133735
DEK	7913	1.232123959
DDX1	1653	1.232106106
SREBF1	6720	1.232098239
HSD17B4	3295	1.232096685
SRP14	6727	1.232069943
UBE4B	10277	1.232046713
SKA2	348235	1.232046713
ANP32A	8125	1.232036606
PPP2R2A	5520	1.231924633
BAIAP2L1	55971	1.231864397
C19orf47	126526	1.230817842
SNTB2	6645	1.230203013
NIF3L1	60491	1.229587923
TRPT1	83707	1.229587923
FKBP9	11328	1.229554304
ZNF33B	7582	1.227124934
GATA4	2626	1.225891862
RFC5	5985	1.225891862
UBE2A	7319	1.225891862
PCNXL2	80003	1.225891862
C3orf63	23272	1.22527493
COCH	1690	1.224657734
PABPC4	8761	1.224125608
CBLL1	79872	1.224040274
EIF2S2	8894	1.223198847
DNMBP	23268	1.222890641
TEAD3	7005	1.222804561
ACTR3C	653857	1.222804561
ARHGEF18	23370	1.222798711
HEBP2	23593	1.221567789
KTN1	3895	1.221117464
ZNFX1	57169	1.220615834
RSF1	51773	1.220329955
TSPAN3	10099	1.21971064
ZNF275	10838	1.21971064
REV3L	5980	1.219091058
PMEPA1	56937	1.219018525
ARHGAP23	57636	1.218519412
RBMS1	5937	1.217851097
CD63	967	1.217240394
KIAA0100	9703	1.217086831
AP3M1	26985	1.217064742
LOXL4	84171	1.21701476
G0S2	50486	1.216977827

Gene	Entrez Gene ID	Log ratio RPKM(IGF2BP3)/RPKM(control IgG)
PRKAR1A	5573	1.216707097
EML4	27436	1.216697149
ZNF395	55893	1.216672987
SEC16A	9919	1.216662431
NFIX	4784	1.216610069
KIF11	3832	1.216602333
PPP2R1B	5519	1.215989154
NUPR1	26471	1.215989154
ACAD9	28976	1.215989154
HIST2H2BE	8349	1.215542768
GTF3C4	9329	1.215367972
SLAIN2	57606	1.215367972
SSFA2	6744	1.21511778
SERBP1	26135	1.214444834
SNAP23	8773	1.214124805
ITGB4	3691	1.213993405
GNAI3	2773	1.212258044
TBL1X	6907	1.211012193
TMEM14E	645843	1.210388864
TMEM138	51524	1.209765266
B4GALT7	11285	1.20851726
HMG20A	10363	1.207892852
TAF4B	6875	1.206643224
STX17	55014	1.206643224
NUP210	23225	1.206183957
C20orf108	116151	1.206018004
DDX18	8886	1.20596812
ARHGAP1	392	1.205965951
TRIM8	81603	1.205709989
RAB22A	57403	1.204766751
RPP38	10557	1.203514411
SLC39A14	23516	1.203457591
CD44	960	1.203320901
TBC1D24	57465	1.202887833
TFRC	7037	1.20268286
CNNM3	26505	1.199750857
PSEN1	5663	1.199122642
COMMD5	28991	1.197865391
TTC38	55020	1.197236355
RNF32	140545	1.195977459
CDC26	246184	1.195977459
DCUN1D2	55208	1.195347598
C5orf51	285636	1.195347598
RECQL5	9400	1.193456366
UBAP1	51271	1.192194165
TNFRSF11B	4982	1.19216022
NGEF	25791	1.191897632
RIC8A	60626	1.191848317
C19orf22	91300	1.191790148

Gene	Entrez Gene ID	Log ratio RPKM(IGF2BP3)/RPKM(control IgG)
RNF167	26001	1.191788475
C19orf63	284361	1.191725285
PPIL1	51645	1.191722081
DMPK	1760	1.191720408
CNOT7	29883	1.191707146
HPS6	79803	1.191707146
NOP14	8602	1.191654349
RABEP1	9135	1.191653302
ATP6V1G1	9550	1.191646558
MTA1	9112	1.191641697
POLR2C	5432	1.191632791
GPT2	84706	1.191615521
CHCHD3	54927	1.191612604
JUNB	3726	1.191608698
FAM175B	23172	1.191584922
TRAPPC4	51399	1.191583466
ARHGAP5	394	1.191562651
FMR1	2332	1.191562651
PRKRIP1	79706	1.191562651
UHMK1	127933	1.191531458
LUC7L2	51631	1.191490628
CKAP4	10970	1.191479795
STX1A	6804	1.191478371
NAA10	8260	1.191472907
COX5B	1329	1.191454244
ECT2	1894	1.191451575
DCTN1	1639	1.191438416
SGTA	6449	1.191391561
MTIF2	4528	1.191376643
SLC10A3	8273	1.191365526
SIPA1	6494	1.191352688
GRPEL1	80273	1.19131258
ATP13A3	79572	1.191292999
DNAJC11	55735	1.191283402
MAF1	84232	1.191282215
CAND1	55832	1.191161856
OCIAD2	132299	1.191161469
SGSM2	9905	1.191108884
TOR3A	64222	1.191017248
HEXA	3073	1.190990322
VPS53	55275	1.190974325
BAG4	9530	1.19093086
TIMM23	10431	1.190854022
TACC3	10460	1.19083976
FAM83G	644815	1.190828463
CSTF2	1478	1.190801302
LIG3	3980	1.190298792
SCGB1D2	10647	1.189033824
SCD	6319	1.187970592

Gene	Entrez Gene ID	Log ratio RPKM(IGF2BP3)/RPKM(control IgG)
EPM2AIP1	9852	1.187767747
NHS	4810	1.186500558
ZNF195	7748	1.186500558
TPCN1	53373	1.186500558
LMNB2	84823	1.185505704
KRCC1	51315	1.185232254
MCL1	4170	1.184789949
ATP2A2	488	1.183735358
SH2D4A	63898	1.182736971
UBE2H	7328	1.182056609
FAM53C	51307	1.182056609
SBNO1	55206	1.182056609
CASP8AP2	9994	1.18142064
USP22	23326	1.180652369
NCOA2	10499	1.180147861
TMC7	79905	1.180147861
SCARB1	949	1.179763194
PHF12	57649	1.178697751
ACP2	53	1.178236585
BUD13	84811	1.176960992
STAT1	6772	1.176437545
MLF1	4291	1.176322773
SLCO4A1	28231	1.175713406
LBR	3930	1.175653354
PLK1	5347	1.175221803
NSUN5P2	260294	1.173767068
ANKFY1	51479	1.172487516
GSK3B	2932	1.171847314
PHACTR2	9749	1.171847314
UBE2Z	65264	1.170557304
NUP98	4928	1.169925001
DYNLT3	6990	1.169283661
NUB1	51667	1.169283661
TGFB1I1	7041	1.168642036
TIMP2	7077	1.168199404
QTRTD1	79691	1.167357928
ALDH7A1	501	1.166715445
CDC42BPA	8476	1.166419058
VAV3	10451	1.166072676
PFKM	5213	1.165071033
SLC38A2	54407	1.164918981
FADS3	3995	1.164786278
ORMDL2	29095	1.164142649
TULP4	56995	1.164142649
NOS1AP	9722	1.163498732
MAP7D3	79649	1.162854528
C11orf82	220042	1.162854528
RAB31	11031	1.161996105
ERCC5	2073	1.161883048

Gene	Entrez Gene ID	Log ratio RPKM(IGF2BP3)/RPKM(control IgG)
CCNT1	904	1.16187316
TMEM167A	153339	1.161856563
ALDH18A1	5832	1.16166096
GPR56	9289	1.161640166
SEC63	11231	1.161571723
UBE3C	9690	1.161512762
RBM12B	389677	1.161414859
MINK1	50488	1.160972794
CPD	1362	1.160274831
NR5A2	2494	1.160274831
GOLPH3	64083	1.159629186
GIPC1	10755	1.159188257
ZNF192	7745	1.15704371
GLI4	2738	1.156396617
PTPN13	5783	1.155101558
ADAM15	8751	1.154986684
MED6	10001	1.154453593
CTR9	9646	1.153668062
STC1	6781	1.153350777
CHST11	50515	1.153145047
PFDN4	5203	1.152507948
PAQR4	124222	1.152507948
INTS3	65123	1.151858817
MLLT6	4302	1.151715847
SF1	7536	1.150937194
FCHSD2	9873	1.150559677
ARL4C	10123	1.150409988
HK2	3099	1.149624536
GSPT1	2935	1.149366552
CENPA	1058	1.149259365
GGA1	26088	1.14860877
NDFIP1	80762	1.14860877
NAV1	89796	1.14860877
PRIC285	85441	1.148487831
PFKP	5214	1.147803674
SMEK2	57223	1.14764819
HCCS	3052	1.147306699
C6orf89	221477	1.147306699
HPDL	84842	1.146655222
C19orf70	125988	1.146655222
DDX6	1656	1.145987331
SPATA2	9825	1.145351386
CABYR	26256	1.145351386
GLB1L2	89944	1.145351386
CMTM4	146223	1.14404637
RFK	55312	1.143393419
ADSL	158	1.143124942
RANBP2	5903	1.142740172
CABIN1	23523	1.142740172

Gene	Entrez Gene ID	Log ratio RPKM(IGF2BP3)/RPKM(control IgG)
CRAMP1L	57585	1.142740172
TSHZ1	10194	1.14208663
MCM10	55388	1.14208663
LRCH3	84859	1.14208663
GADD45A	1647	1.141432791
CARHSP1	23589	1.140778656
GTPBP8	29083	1.140778656
NR3C1	2908	1.140124224
KCTD3	51133	1.139906857
ANAPC5	51433	1.139859938
RNF41	10193	1.139535489
DDX41	51428	1.139466789
SDCCAG8	10806	1.13926347
LOC654433	654433	1.139228204
STK38	11329	1.139228204
TESK1	7016	1.139203717
CKAP2L	150468	1.139189408
DHRS3	9249	1.139188474
IDH2	3418	1.13917331
ERF	2077	1.139157242
TAF9	6880	1.139120342
C9orf167	54863	1.139118571
NSMAF	8439	1.139084188
UBE2C	11065	1.139064038
PRSS23	11098	1.139064038
AGAP1	116987	1.138976413
PDLIM5	10611	1.138976413
GPR39	2863	1.138956023
SLC25A1	6576	1.138944623
B3GALT5	10317	1.138857611
STX16	8675	1.138839607
C3orf58	205428	1.138816656
PRODH	5625	1.138712794
TBL1XR1	79718	1.138662781
FARSB	10056	1.13865354
CHPF2	54480	1.13865354
GLUL	2752	1.138550282
SH3BP5L	80851	1.138527801
PCSK5	5125	1.13847391
RAB11FIP3	9727	1.138431932
PHF20L1	51105	1.138159145
HYLS1	219844	1.138159145
RELB	5971	1.136847604
HECTD3	79654	1.136191386
C14orf109	26175	1.13553487
LRRC16A	55604	1.1344502
TMEM39B	55116	1.13422094
GADD45B	4616	1.132905812
PGGT1B	5229	1.132905812

Gene	Entrez Gene ID	Log ratio RPKM(IGF2BP3)/RPKM(control IgG)
ADAT1	23536	1.132247798
XRRA1	143570	1.132247798
SEPT10	151011	1.131589484
LRPPRC	10128	1.131399883
EIF3H	8667	1.131394051
GAS8	2622	1.13093087
DDX49	54555	1.13093087
SEPX1	51734	1.129612738
RXRA	6256	1.129572342
FAM3C	10447	1.129529821
TCTN3	26123	1.126972856
KARS	3735	1.126681335
MYL12B	103910	1.12659757
ACSL4	2182	1.125651102
TMEM63B	55362	1.12498977
CCDC77	84318	1.12498977
HTATIP2	10553	1.122341408
PIGU	128869	1.122341408
HIST1H3C	8352	1.121678557
B3GNT5	84002	1.121678557
EPT1	85465	1.121471464
SLC7A5	8140	1.121310199
ADD1	118	1.121286288
TTF2	8458	1.121226183
SET	6418	1.121121019
EIF2S3	1968	1.121002867
EREG	2069	1.120993623
DENND1A	57706	1.12035194
ZNF592	9640	1.120319973
GNPDA1	10007	1.119688175
LSM1	27257	1.119688175
PPP2R2D	55844	1.119688175
MALAT1	378938	1.119170803
UPP1	7378	1.117695043
SNRNP35	11066	1.117030053
MANSC1	54682	1.117030053
ETV6	2120	1.116364757
UBR5	51366	1.115989213
DSCAML1	57453	1.115699153
ARFGAP2	84364	1.115033243
LOC441454	441454	1.115033243
ST3GAL1	6482	1.114761639
CSDA	8531	1.114718902
NFX1	4799	1.114367025
HNRNPH2	3188	1.114142566
HYOU1	10525	1.113956189
SFXN4	119559	1.113033665
ABTB2	25841	1.112366523
AIG1	51390	1.112366523

Gene	Entrez Gene ID	Log ratio RPKM(IGF2BP3)/RPKM(control IgG)
EHBP1L1	254102	1.112321829
FAM129B	64855	1.111948328
UNC119	9094	1.111031312
LMBRD1	55788	1.111031312
METAP1	23173	1.110363243
DCAF6	55827	1.110363243
DHRS7	51635	1.109026176
PCYT2	5833	1.107687869
SVIP	258010	1.107687869
VDAC1	7416	1.107280906
LOC90784	90784	1.107248061
EAF1	85403	1.107233223
CBX3	11335	1.107174316
KIAA0141	9812	1.10701825
XPO5	57510	1.106915204
PLOD3	8985	1.106885072
PI4K2A	55361	1.106872831
SPG7	6687	1.106852824
MED22	6837	1.106758466
CALD1	800	1.106745005
PLEKHA6	22874	1.106740448
RAB11FIP1	80223	1.10669424
WDR74	54663	1.106693932
KDELC1	79070	1.10668069
LRRC41	10489	1.106646665
TMED3	23423	1.106620345
TBC1D10B	26000	1.106560123
C11orf84	144097	1.106518347
CDC6	990	1.106498001
PTOV1	53635	1.106495622
VPS25	84313	1.106480941
CDC20	991	1.106390468
VPS52	6293	1.106377453
ABI2	10152	1.10634832
FTSJ1	24140	1.106302859
CANT1	124583	1.106205507
NLE1	54475	1.106133524
GON4L	54856	1.106052205
GRAMD4	23151	1.106041637
ATP2B4	493	1.106031903
VTA1	51534	1.105678078
SKIV2L	6499	1.105007525
RRP7B	91695	1.103665483
DTNBP1	84062	1.102993993
PRR22	163154	1.102993993
RPL4	6124	1.102944132
CELF1	10658	1.102825491
GPC4	2239	1.101650076
DSCR3	10311	1.101650076

Gene	Entrez Gene ID	Log ratio RPKM(IGF2BP3)/RPKM(control IgG)
AMMECR1L	83607	1.101650076
ZSWIM1	90204	1.101650076
NDUFV2	4729	1.100977648
GNAI2	2771	1.10095199
NACC1	112939	1.100646332
ZBTB11	27107	1.09895848
LTBR	4055	1.098540969
CUX1	1523	1.098299607
CSDE1	7812	1.097141158
PHF8	23133	1.096936483
IVNS1ABP	10625	1.096261853
STRA6	64220	1.096261853
TUBA1C	84790	1.095860015
GRIPAP1	56850	1.095614395
OSGIN2	734	1.095586908
EPN2	22905	1.095586908
GABARAPL1	23710	1.095586908
GBF1	8729	1.094982418
HSPA4L	22824	1.094911647
STAT6	6778	1.094634165
MCM3AP	8888	1.094486867
PAK2	5062	1.094467468
CLEC16A	23274	1.09443969
E2F4	1874	1.094207571
CCDC88A	55704	1.094010024
TNK2	10188	1.093976148
HIVEP1	3096	1.093560176
ZNF786	136051	1.093560176
OLFML2A	169611	1.093560176
ELF1	1997	1.092883966
MTRR	4552	1.092883966
KIAA1143	57456	1.092883966
ZNF37A	7587	1.092207438
ZNHIT6	54680	1.091530593
MAP2K4	6416	1.09085343
SNRK	54861	1.089498151
SMC1A	8243	1.089435084
CLCC1	23155	1.088820033
THYN1	29087	1.088820033
UPF3B	65109	1.088820033
IKBIP	121457	1.08819215
AKR1A1	10327	1.088141597
ZSWIM7	125150	1.088141597
MAPK3	5595	1.086299846
STAG1	10274	1.085424656
GAK	2580	1.08519465
SNX19	399979	1.085011937
USP1	7398	1.084900411
USP33	23032	1.084869231

Gene	Entrez Gene ID	Log ratio RPKM(IGF2BP3)/RPKM(control IgG)
RPL41	6171	1.084794136
TBRG4	9238	1.084748209
GRWD1	83743	1.084731498
SERP1	27230	1.084728375
NPC1	4864	1.084718356
EHMT1	79813	1.084715388
SGPL1	8879	1.084655338
VASP	7408	1.084472007
IKBKB	3551	1.084413205
M6PR	4074	1.084392187
FUBP3	8939	1.084345588
LRBA	987	1.084064265
TGFBR2	7048	1.084064265
ZBTB45	84878	1.084064265
LATS1	9113	1.083958344
PTK2B	2185	1.083275044
MGAT1	4245	1.083051376
C6orf106	64771	1.082323045
ZDHHC16	84287	1.081300102
TMEM41A	90407	1.080657663
PARP2	10038	1.079292767
ADM2	79924	1.079292767
RBMX	27316	1.078916468
MTOR	2475	1.078824268
PHF15	23338	1.078560536
ZNF330	27309	1.077926579
EIF5	1983	1.077465415
CNN2	1265	1.076408865
CLINT1	9685	1.07599672
DPYSL2	1808	1.075917127
IRF7	3665	1.075874867
KCNK5	8645	1.075874867
NRM	11270	1.075874867
NUDT2	318	1.075190314
VCAN	1462	1.074685724
ASPM	259266	1.073820233
RPRD1A	55197	1.07244885
USP54	159195	1.07244885
RAP1GAP2	23108	1.072126757
PKD1	5310	1.071567519
SEPT9	10801	1.071034252
FGD1	2245	1.069702167
ZNF138	7697	1.069702167
COMMD10	51397	1.069702167
TMEM104	54868	1.069702167
TACC2	10579	1.068919065
TUBGCP2	10844	1.068908501
NSF	4905	1.068890903
F3	2152	1.068779164

Gene	Entrez Gene ID	Log ratio RPKM(IGF2BP3)/RPKM(control IgG)
SEPT2	4735	1.068634903
RAI1	10743	1.068616612
EIF6	3692	1.068559657
VDAC2	7417	1.06853608
SCARB2	950	1.068511564
DKFZp761E198	91056	1.068503564
MTHFD2	10797	1.068450448
PFN1	5216	1.068422181
NCKAP1	10787	1.068386975
TMED5	50999	1.068341499
TBXAS1	6916	1.068326861
MTMR4	9110	1.067971858
SSR4	6748	1.067874479
RAB2B	84932	1.066950244
SNHG3	8420	1.065916918
BCAN	63827	1.065572312
AKIRIN1	79647	1.065445665
KIN	22944	1.064882852
SPNS2	124976	1.064373912
MAPK14	1432	1.064193062
GAMT	2593	1.062812492
LAS1L	81887	1.062491842
UBR4	23352	1.062400507
FA2H	79152	1.062326227
RALY	22913	1.062248692
UBE2D2	7322	1.062121712
C12orf41	54934	1.062121712
RRAGC	64121	1.062121712
MTAP	4507	1.061845752
ZFAND5	7763	1.0614306
LOC399744	399744	1.060739158
ARNT	405	1.060047384
GLTSCR1	29998	1.059355278
TOE1	114034	1.059355278
CARD10	29775	1.059000591
MBP	4155	1.057754011
PIGS	94005	1.057276965
NOM1	64434	1.056997561
RXRB	6257	1.056583528
BAZ2B	29994	1.056583528
ARV1	64801	1.056583528
ZC3H15	55854	1.0564456
KIAA0226	9711	1.056124875
AP1G2	8906	1.055889758
LARP7	51574	1.055833889
ZBTB1	22890	1.055195654
MKL2	57496	1.055195654
SRF	6722	1.054501216
TIAM1	7074	1.054501216

Gene	Entrez Gene ID	Log ratio RPKM(IGF2BP3)/RPKM(control IgG)
SHPK	23729	1.054501216
KIAA1244	57221	1.053806444
TAS2R30	259293	1.053806444
COL17A1	1308	1.053210921
BCAR3	8412	1.053063738
EXT1	2131	1.052415894
NUS1	116150	1.052415894
FAM149A	25854	1.051720116
FCF1	51077	1.051720116
NCEH1	57552	1.051636959
GOLGB1	2804	1.05126867
CMTM6	54918	1.051024003
UNC119B	84747	1.051024003
UMPS	7372	1.050327554
EFR3A	23167	1.050327554
BBS1	582	1.049630768
ADAM9	8754	1.049309775
AZI2	64343	1.048933645
DSN1	79980	1.048236186
CD46	4179	1.04822121
ZSWIM4	65249	1.047538389
TIGD5	84948	1.047538389
TOX2	84969	1.047538389
FARSA	2193	1.04735277
DNAJB1	3337	1.047214971
ANXA1	301	1.047128076
CTDSP2	10106	1.047025335
HNRNPA3	220988	1.04701067
NFKBIZ	64332	1.046961369
AGPAT6	137964	1.046921047
QRICH1	54870	1.046859589
CPSF2	53981	1.046844863
SSH2	85464	1.046840254
RBCK1	10616	1.046815014
HIP1	3092	1.046141782
TRIM2	23321	1.046141782
SNHG7	84973	1.044743821
CLK1	1195	1.044044333
WASF3	10810	1.044044333
PSMD5	5711	1.043344505
PITPNM1	9600	1.043152138
FOSB	2354	1.042661152
BAG5	9529	1.04194383
STRADA	92335	1.041118987
EBF3	253738	1.039840265
GNL3	26354	1.039629922
WDR36	134430	1.039629376
GALNT2	2590	1.039579601
AGPAT3	56894	1.039577658

Gene	Entrez Gene ID	Log ratio RPKM(IGF2BP3)/RPKM(control IgG)
SLC2A4RG	56731	1.039467316
POLR2K	5440	1.039138394
TNFRSF10B	8795	1.038168762
SPOP	8405	1.037030731
C1RL	51279	1.037030731
CDCA4	55038	1.035956878
PHF2	5253	1.03562391
DPY19L1	23333	1.034919984
MLLT4	4301	1.03487046
SMARCA4	6597	1.034838779
DVL1	1855	1.034518941
CHMP4B	128866	1.03355256
NBN	4683	1.033545716
SRA1	10011	1.033511102
NMT1	4836	1.033259767
WBP11	51729	1.033178599
AGBL5	60509	1.032806145
DAPK1	1612	1.031816447
CBL	867	1.029982866
DDR1	780	1.029345875
SAMD9	54809	1.029276182
AP1G1	164	1.028569152
PEA15	8682	1.028272073
BCLAF1	9774	1.028030871
DPP9	91039	1.027946541
PPFIA1	8500	1.027911351
PTDSS2	81490	1.027861775
SMCR8	140775	1.027861775
GNAS	2778	1.027836564
DHRS9	10170	1.027154052
CDC14A	8556	1.02644598
WWC2	80014	1.02644598
BAZ1B	9031	1.025766164
TACO1	51204	1.025737561
GPR180	160897	1.024319679
LOC388789	388789	1.024319679
ARF1	375	1.024237101
KRT6A	3853	1.023610215
TRMU	55687	1.02219024
THRAP3	9967	1.021688132
JUP	3728	1.021576851
ZNF143	7702	1.020768865
SOCS5	9655	1.020768865
PCBP4	57060	1.020768865
MYO10	4651	1.020730682
SRP72	6731	1.019867317
ABL1	25	1.019757693
FER	2241	1.019346089
NARF	26502	1.019346089

Gene	Entrez Gene ID	Log ratio RPKM(IGF2BP3)/RPKM(control IgG)
MOCS3	27304	1.019346089
TUBGCP5	114791	1.019346089
ZBED4	9889	1.018634174
UNC13A	23025	1.018634174
FZD4	8322	1.017921908
ASPHD1	253982	1.017921908
ITGB1	3688	1.016768749
AFG3L2	10939	1.012926174
ASNSD1	54529	1.012211084
ZNF673	55634	1.012211084
PILRB	29990	1.011681805
IGHMBP2	3508	1.011495639
HNRNPD	3184	1.011039076
TRAF7	84231	1.010959542
ARG2	384	1.010779839
NCRNA00152	112597	1.010779839
LIPH	200879	1.010779839
SUPV3L1	6832	1.010063683
KIF18A	81930	1.010063683
LDLRAD3	143458	1.010063683
AFAP1L2	84632	1.008630305
GNL3L	54552	1.00830446
C10orf26	54838	1.008271408
REPS1	85021	1.007913082
PAGE2B	389860	1.007913082
ACRV1	56	1.005040617
STRN	6801	1.004321606
SERPINA6	866	1.003602237
PRICKLE3	4007	1.002162421
KIF1C	10749	1.001783779
POLE3	54107	1.001603886
MED12	9968	1.001441974
HAUS4	54930	1.000721167
ANXA4	307	1.000659216

Table S2: GO analysis of the IGF2BP3-bound mRNAs

Accession	GO Term	Ratio (Numerator)	Ratio (Denominator)	P-value
GO:0006915	apoptosis	145	582	2.27E-48
GO:0007049	cell cycle	108	403	1.96E-39
GO:0006468	protein phosphorylation	114	487	1.68E-35
GO:0007165	signal transduction	185	1147	3.46E-33
GO:0051301	cell division	74	262	4.97E-29
GO:0007264	small GTPase mediated signal transduction	74	293	1.09E-25
GO:0000278	mitotic cell cycle	76	308	1.19E-25
GO:0007173	epidermal growth factor receptor signaling pathway	30	75	2.58E-17
GO:0008624	induction of apoptosis by extracellular signals	34	104	2.92E-16
GO:0008283	cell proliferation	60	302	9.24E-16
GO:0051056	regulation of small GTPase mediated signal transduction	42	169	5.94E-15
GO:0008285	negative regulation of cell proliferation	59	308	8.84E-15
GO:0006917	induction of apoptosis	38	175	9.88E-12
GO:0007050	cell cycle arrest	31	126	2.66E-11
GO:0008286	insulin receptor signaling pathway	32	138	6.82E-11
GO:0008284	positive regulation of cell proliferation	53	326	1.34E-10
GO:0042981	regulation of apoptosis	37	182	1.38E-10
GO:0035023	regulation of Rho protein signal transduction	22	71	1.62E-10
GO:0030036	actin cytoskeleton organization	29	121	2.26E-10
GO:0007265	Ras protein signal transduction	21	66	2.41E-10
GO:0007266	Rho protein signal transduction	16	38	2.76E-10
GO:0030154	cell differentiation	69	496	4.15E-10
GO:0002224	toll-like receptor signaling pathway	21	72	1.46E-09
GO:0043066	negative regulation of apoptosis	36	189	1.59E-09
GO:0007160	cell-matrix adhesion	21	73	1.93E-09
GO:0035556	intracellular signal transduction	43	256	2.58E-09
GO:0051726	regulation of cell cycle	18	55	2.75E-09
GO:0048015	toll-like receptor 1 signaling pathway	20	69	4.02E-09
GO:0034130	toll-like receptor 2 signaling pathway	20	69	4.02E-09
GO:0034134	phosphatidylinositol-mediated signaling	20	69	4.02E-09
GO:0007243	intracellular protein kinase cascade	23	90	4.24E-09
GO:0008063	Toll signaling pathway	21	76	4.31E-09
GO:0034142	toll-like receptor 4 signaling pathway	21	76	4.31E-09
GO:0034138	toll-like receptor 3 signaling pathway	19	63	4.71E-09
GO:0043065	positive regulation of apoptosis	29	138	5.91E-09
GO:0006916	anti-apoptosis	35	191	7.63E-09
GO:0035329	hippo signaling cascade	9	13	7.64E-09
GO:0008219	cell death	29	141	9.89E-09

Accession	GO Term	Ratio (Numerator)	Ratio (Denominator)	P-value
GO:0030335	positive regulation of cell migration	21	83	2.39E-08
GO:0045786	negative regulation of cell cycle	14	39	4.16E-08
GO:0007155	cell adhesion	68	542	4.38E-08
GO:0002756	MyD88-independent toll-like receptor signaling pathway	18	65	5.22E-08
GO:0006511	ubiquitin-dependent protein catabolic process	29	152	5.69E-08
GO:0000082	G1/S transition of mitotic cell cycle	27	135	5.82E-08
GO:0043547	positive regulation of GTPase activity	25	119	6.48E-08
GO:0008654	phospholipid biosynthetic process	16	53	7.55E-08
GO:0034329	cell junction assembly	21	89	8.85E-08
GO:0007166	cell surface receptor linked signaling pathway	30	167	1.37E-07
GO:0006921	cellular component disassembly involved in apoptosis	15	49	1.57E-07
GO:0042127	regulation of cell proliferation	19	78	2.10E-07
GO:0043123	positive regulation of I-kappaB kinase/NF-kappaB cascade	26	135	2.23E-07
GO:0042059	negative regulation of epidermal growth factor receptor signaling pathway	12	33	3.30E-07
GO:0016477	cell migration	18	75	5.53E-07
GO:0008645	MAPKKK cascade	13	42	9.09E-07
GO:0008633	activation of pro-apoptotic gene products	11	31	1.36E-06
GO:0007229	integrin-mediated signaling pathway	15	57	1.37E-06
GO:0030032	lamellipodium assembly	8	16	1.78E-06
GO:0008629	induction of apoptosis by intracellular signals	12	40	3.45E-06
GO:0042060	wound healing	14	54	3.71E-06
GO:0040008	regulation of growth	14	55	4.69E-06
